# Glycerol Hypersensitivity in a *Drosophila* Model for Glycerol Kinase Deficiency Is Affected by Mutations in Eye Pigmentation Genes

**DOI:** 10.1371/journal.pone.0031779

**Published:** 2012-03-09

**Authors:** Patrick J. Wightman, George R. Jackson, Katrina M. Dipple

**Affiliations:** 1 Department of Human Genetics, David Geffen School of Medicine at University of California Los Angeles, Los Angeles, California, United States of America; 2 Department of Neurology, David Geffen School of Medicine at University of California Los Angeles, Los Angeles, California, United States of America; 3 Brain Research Institute, Semel Institute for Neuroscience and Human Behavior, David Geffen School of Medicine at University of California Los Angeles, Los Angeles, California, United States of America; 4 Center for Neurobehavioral Genetics, Semel Institute for Neuroscience and Human Behavior, David Geffen School of Medicine at University of California Los Angeles, Los Angeles, California, United States of America; 5 Department of Pediatrics, David Geffen School of Medicine at University of California Los Angeles, Mattel Children's Hospital at University of California Los Angeles, Los Angeles, California, United States of America; Thomas Jefferson University, United States of America

## Abstract

Glycerol kinase plays a critical role in metabolism by converting glycerol to glycerol 3-phosphate in an ATP dependent reaction. In humans, glycerol kinase deficiency results in a wide range of phenotypic variability; patients can have severe metabolic and CNS abnormalities, while others possess hyperglycerolemia and glyceroluria with no other apparent phenotype. In an effort to help understand the pathogenic mechanisms underlying the phenotypic variation, we have created a *Drosophila* model for glycerol kinase deficiency by RNAi targeting of *dGyk* (CG18374) and *dGK* (CG7995). As expected, RNAi flies have reduced glycerol kinase RNA expression, reduced phosphorylation activity and elevated glycerol levels. Further investigation revealed these flies to be hypersensitive to fly food supplemented with glycerol. Due to the hygroscopic nature of glycerol, we predict glycerol hypersensitivity is a result of greater susceptibility to desiccation, suggesting glycerol kinase to play an important role in desiccation resistance in insects. To evaluate a role for genetic modifier loci in determining severity of the glycerol hypersensitivity observed in knockdown flies, we performed a preliminary screen of lethal transposon insertion mutant flies using a glycerol hypersensitive survivorship assay. We demonstrate that this type of screen can identify both enhancer and suppressor genetic loci of glycerol hypersensitivity. Furthermore, we found that the glycerol hypersensitivity phenotype can be enhanced or suppressed by null mutations in eye pigmentation genes. Taken together, our data suggest proteins encoded by eye pigmentation genes play an important role in desiccation resistance and that eye pigmentation genes are strong modifiers of the glycerol hypersensitive phenotype identified in our *Drosophila* model for glycerol kinase deficiency.

## Introduction

In this study, we use *Drosophila* as a model organism for the study of glycerol kinase deficiency (GKD [MIM 307030]). The metabolic role of glycerol kinase is to convert glycerol to glycerol 3-phosphate in an ATP-dependent reaction and is the rate-limiting step in glycerol utilization [1]. Glycerol 3-phosphate can be directed towards gluconeogenesis or lipid metabolism and alteration of GK activity also has a substantial effect on metabolic flux through other metabolic pathways such as the pentose phosphate pathway [2]. In humans, GKD patients can have severe metabolic and CNS abnormalities, while others possess hyperglycerolemia and glyceroluria with no other apparent phenotype [3], [4]. Extensive studies incorporating patient data, mutation analysis and protein tertiary structure reveal no obvious phenotype-genotype correlations [4]–[6]. Additionally, analysis of glycerol kinase activity in GKD patients shows a range of glycerol kinase (GK) activities that do not correspond to severity of the phenotype [4]. The cause of the phenotypic variability in GKD is currently unknown.

It has previously been hypothesized that glycerol kinase could possess alternative functions [4] *i.e.* protein activities. This is supported by the identification of rat GK as an ATP stimulated glucocorticoid-receptor translocation promoter protein [7], [8]. Additionally, evidence for an apoptotic function of glycerol kinase has been identified by weighted gene co-expression network analysis of liver gene expression in glycerol kinase knockout mice liver gene expression [9]. In addition to these alternative activities, it has been proposed that modifier loci could influence the GKD phenotype severity [4], [10]–[12]. Our aim in this study was to create a model to study GKD and access the power of *Drosophila* genetics to dissect the underlying complex pathogenic mechanism.

Animal models for human diseases can provide insights into pathogenic mechanisms of disease that cannot be deduced from patient studies. For example, analysis of adipose tissue from glycerol kinase knockout mice revealed altered expression levels of genes involved in the insulin signaling pathway in addition to lipid and carbohydrate metabolism [13], [14]. However, glycerol kinase knockout mice die at postnatal day 3 or 4, making this a challenging animal model to study [15], [16]. *Drosophila* is an alternative animal model and possesses a wide array of classical and molecular genetic techniques available for investigating gene function [17], [18]. Analysis of the *Drosophila melanogaster* genome sequence reveals the presence of all the genes encoding enzymes involved in glycerol metabolism in humans [19]. There are five glycerol kinase-related genes, only two of which are predicted using *in silico* analysis to possess phosphorylation activity (*dGyk* (CG18374) and *dGK* (CG7995)). In addition to the “FGGY” carbohydrate kinase domain that both dGyk and dGK possess [20], [21], amino acid sequence analysis reveals several protein domains with putative roles in protein interaction and mitochondrial apoptosis [19]. This suggests the *Drosophila* glycerol kinase proteins could possess novel alternative protein functions.

Using the *UAS*-GAL4 system for RNAi-mediated knockdown of gene expression in *Drosophila* [22]–[24], we have successfully targeted *dGyk* and *dGK* to create a *Drosophila* model for GKD. Ubiquitous expression of the RNAi constructs results in decreased glycerol kinase RNA expression and reduced GK enzymatic activity. As expected glycerol levels were found to be elevated.

Investigation of knockdown flies identified a glycerol hypersensitive phenotype when fed a glycerol only food source, which we predict to be due to increased susceptibility to desiccation. The control of metabolite composition plays an important role in water balance and is critical for insect survival [25] especially in arid conditions [26]. Additionally, control of glycerol levels through aquaporins is known to play an important role in desiccation tolerance in larvae of the goldenrod gall fly, *Eurosta solidaginis* [27]. Therefore we suspect glycerol hypersensitivity is due to a combination of altered glycerol levels in the RNAi knockdown flies in addition to the hygroscopic nature of glycerol in the fly food.

We adapted the glycerol hypersensitive phenotype to create a glycerol hypersensitive survivorship assay to perform a preliminary screen of lethal transposon insertion mutants with the aim of identifying enhancers and suppressors the glycerol hypersensitive phenotype. From this screen, we are able to identify both enhancers and suppressors of glycerol hypersensitivity including one synthetic lethal cross. We also found a strong effect on glycerol hypersensitivity by eye pigmentation null mutations. Therefore our data reveal a novel link between glycerol kinase and eye pigmentation genes and suggests a novel role for these proteins in desiccation resistance.

## Results

### Creation of a *Drosophila* model for glycerol kinase deficiency

In this study, we used the UAS-GAL4 system [23] for RNAi-mediated knockdown of *dGyk* and *dGK* expression. Inverted repeats (IR) for both *dGyk* and *dGK* were cloned into the *pUDsGFP* plasmid [28] and the resulting transgenic RNAi *Drosophila* lines generated were named *dGyk*-IR and *dGK*-IR. For over-expression lines, complete open reading frames for *dGyk* and *dGK* were subcloned into the *pEX*-UAS vector and named *dGyk*-OE and *dGK*-OE respectively. All *dGyk-* and *dGK*-related fly lines (RNAi, over-expression, P element insertions) are listed in Table S1.

Initial analysis was performed using a *Tubulin*-GAL4 (*Tub*-GAL4) driver for ubiquitous expression of the inserted construct. For RNAi fly lines, this involved setting up crosses between each RNAi fly line with the *Tub*-GAL4 driver flies (9× *dGyk*-IR and 10× *dGK*-IR). Similarly, each over-expression fly line was crossed to the *Tub*-GAL4 driver flies (7× *dGyk*-OE and 7× *dGK*-OE). Progeny from each cross were examined for physical phenotypes. Analysis of *dGyk*-IR×*Tub*-GAL4 crosses revealed 3 lines that resulted in viable adults flies and 6 lines that resulted in progeny that died during larval development. For *dGK*-IR×*Tub*-GAL4 crosses, 8 lines resulted in viable adults flies and 2 lines resulted in progeny that died during larval development.

To determine the basis of lethality, we performed western blot analysis for GFP in knockdown roaming 3^rd^ instar larvae (the *pUds*GFP RNAi vector co-expresses GFP). This would provide an indirect measure of the inverse repeat (IR) expression levels, for example greater GFP levels would indicate greater levels IR expression and infer greater knockdown of the target gene expression levels. For *dGyk*-IR; *Tub*-GAL4 larvae, western blot analysis revealed higher GFP levels in knockdown 3^rd^ instar larvae that died before eclosion than in 3^rd^ instar larvae than developed into glycerol hypersensitive adult flies (Figure S1 and Methods S1). A similar trend was observed for *dGK*-IR; *Tub*-GAL4 3^rd^ instar larvae. Therefore larval lethality is likely due to lower levels of dGyk and dGK due to greater expression of the *dGyk*-IR and *dGK*-IR construct. Unfortunately, we were unable to identify *Drosophila* dGyk- and dGK-specific antibodies. Both commercially available glycerol kinase antibodies as well as ones designed by us were non-specific for dGyk or dGK.

In this study, we focused on the RNAi lines that produced live adult flies when crossed to the *Tub*-GAL4. The analysis of progeny from *dGyk*-OE×*Tub*-GAL4 crosses produced adult progeny with no physical phenotype. However *dGK*-OE; *Tub*-GAL4 progeny were found to be embryonic lethality. For all subsequent experiments, 2 fly lines for each RNAi phenotype were chosen for analysis (results are shown for single fly lines).

**Figure 1:**
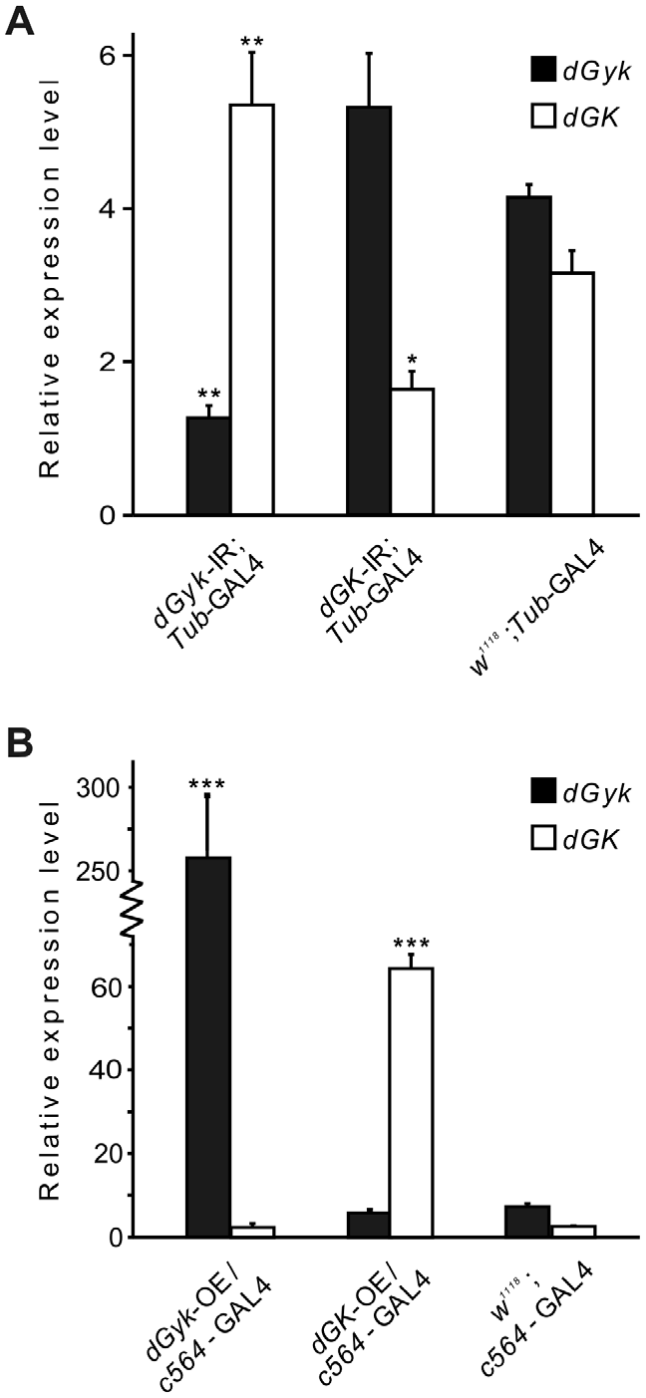
Generation of transgenic flies for RNAi (*dGyk*-IR, *dGK*-IR) and over-expression (*dGyk*-OE, *dGK*-OE) analysis. Inverted repeats (IR) for both *dGyk* and *dGK* were subcloned into the *pUDsGFP* vector [28]. This vector allows expression of the double-stranded (dsRNA) transcripts from the RNAi construct under the control of a *UAS*-binding site for the yeast GAL4 transcription factor. (A) RNA expression levels were determined by qRT-PCR for *dGyk*-IR and *dGK*-IR 3^rd^ instar progeny (using a *Tubulin*-GAL4 driver). *dGyk*-IR/*Tub*-GAL4 had reduced expression of *dGyk* and *dGK*-IR/*Tub*-GAL4 progeny had reduced expression of *dGK*. RNA levels for parental construct fly lines were also determined but were not significantly different to the *w^1118^*; *Tub*-GAL4 control (Figures S2A and S2B). (B) For transcript over-expression (OE) studies, cDNA fragments covering the entire coding regions for *dGyk* and *dGK* were subcloned into the *pEX-UAS* vector [54]. Compared to control 3^rd^ instar larvae, both *c564*-GAL4; *dGyk*-OE and *c564*-GAL4; *dGK*-OE 3^rd^ instar larvae had increased expression levels for *dGyk* and *dGK* respectively. RNA levels for parental construct fly lines were also determined but were not significantly different to the *w^1118^*; *c564*-GAL4 control (Figure S2C and S2D). Statistical analysis using ANOVA was performed by comparison to GAL4 control fly line. *P<0.05, **P<0.01, ***P<0.001.

Analysis of RNAi progeny from *Tub*-GAL4 crosses by qRT-PCR confirmed RNAi had successfully knocked down expression of *dGyk* and *dGK* (Figure 1A). For over-expression analysis of 3^rd^ instar larvae, a larval fat body GAL4 driver (*c564*-GAL4, [29]) driver was used as this produced live progeny for both *dGyk*-OE and *dGK*-OE. Additionally, expression of glycerol kinase is highest in the human liver [13]. Therefore the *c564*-GAL4 driver is an appropriate GAL4 driver for the study of glycerol kinase as it has previously been shown to drive expression of GAL4 in the larval fat body [29], a tissue that plays an important role in energy metabolism similar to that of mammalian liver [30]. The *c564*-GAL4; *dGyk*-OE and *c564*-GAL4; *dGK*-OE progeny had increased expression for *dGyk* and *dGK* respectively (Figure 1B). In this study, the use of the *dGyk*-OE and *dGK*-OE fly lines was restricted to rescue of phenotype experiments. There was no significant statistical difference between control fly lines (GAL4 driver versus construct-only fly lines) indicating no significant leaky construct expression in either RNAi or over-expression construct lines (Figure S2).

### Reduced glycerol phosphorylation activity and elevated glycerol levels by RNAi knockdown of *dGyk* and *dGK* expression

**Figure 2:**
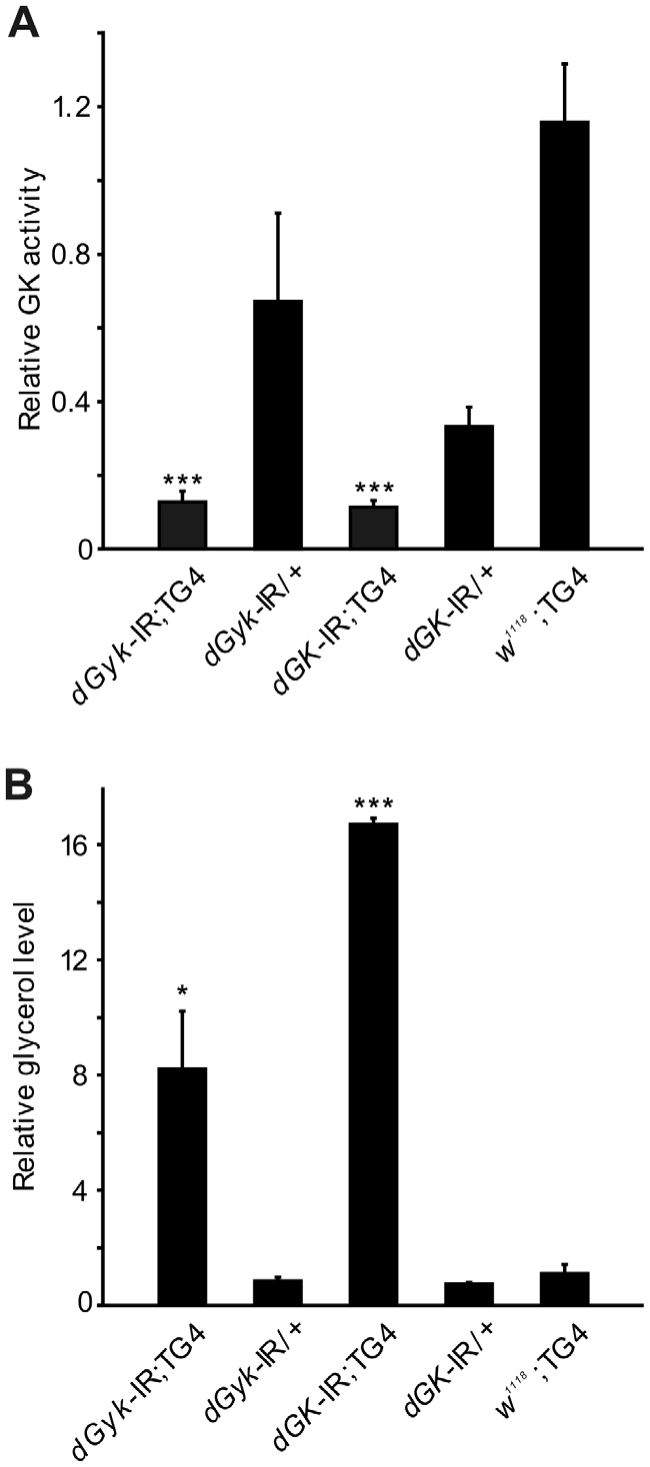
Quantification of glycerol kinase activity and glycerol. (A) Glycerol kinase activity was reduced in both *dGyk*-IR; *Tub*-GAL4 and *dGK*-IR; *Tub*-GAL4 3^rd^ instar larvae compared to both parental controls *w^1118^*; *Tub*-GAL4 and construct control. (Note: *Tub*-GAL4 abbreviated to TG4) (B) Glycerol levels were elevated in both *dGyk*-IR; *Tub*-GAL4 and *dGK*-IR; *Tub*-GAL4 3^rd^ instar larvae compared to parental controls. Error bars represent standard error between biological replicates. Statistical analysis using ANOVA was performed by comparison to parental controls. *P<0.05, **P<0.01, ***P<0.001.

Glycerol kinase phosphorylates glycerol to glycerol 3-phosphate. Using radiolabelled ^14^C glycerol to assay for glycerol kinase (GK) phosphorylation activity, we found reduced GK activity in both *dGyk*-IR; *Tub*-GAL4 and *dGK*-IR; *Tub*-GAL4 3^rd^ instar larval progeny (Figure 2A). With reduced GK activity, we would anticipate elevated glycerol levels. As expected, we found increased levels of glycerol in both *dGyk*-IR; *Tub*-GAL4 and *dGK*-IR; *Tub*-GAL4 3^rd^ instar larvae (Figure 2B). Triglyceride levels in all RNAi progeny were not significantly altered compared to controls (data not shown).

### RNAi targeting of *dGyk* and *dGK* results in glycerol hypersensitive flies

**Figure 3:**
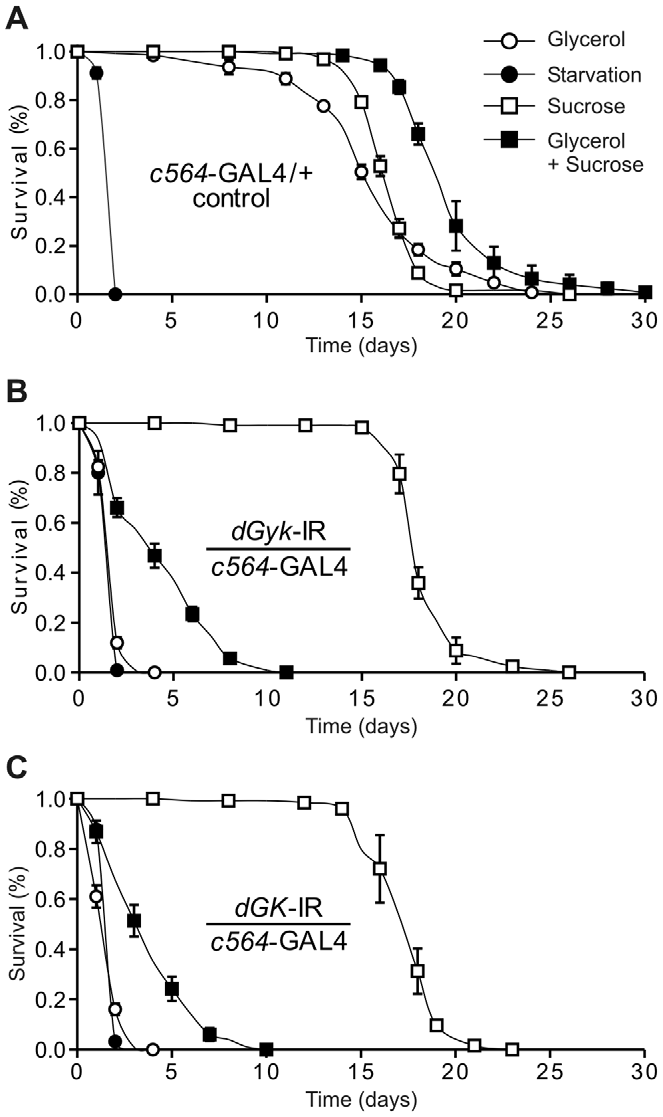
*dGyk*- and *dGK*-knockdown flies are hypersensitive to glycerol. When placed on a glycerol only diet, both *dGyk*-IR/*c564*-GAL4 and *dGK*-IR/*c564*-GAL4 flies died at a similar rate to starvation (control flies were relatively glycerol tolerant). RNAi flies had relatively normal survival on sucrose media compared to controls, but were intolerant to glycerol+sucrose media indicating hypersensitivity to glycerol. Survival analysis of 7-day old male RNAi flies was performed on defined media: glycerol (open circle); starvation (filled circle); sucrose (open square); glycerol+sucrose (filled square). Genotypes tested were (A) control flies, *w^1118^*; *c564*-GAL4/+, (B) *w^1118^*; *dGyk*-IR/*c564*-GAL4, (C) *w^1118^*; *dGK*-IR/*c564*-GAL4. Survivorship assays using parental construct control flies were also performed but were not found to be glycerol hypersensitive. For each genotype and media type, percentage survivorship ± standard error was calculated from 5 vials of 20–25 flies. Survival analysis was performed using the log-rank test on the Kaplan and Meier data.

We hypothesized that reduced GK activity caused by knockdown of *dGyk* or *dGK* expression could affect the ability of *Drosophila* to metabolize glycerol. Therefore we performed survivorship assays using male RNAi knockdown flies on defined food sources: glycerol only, sucrose only, glycerol+sucrose, and agarose (starvation). Control flies were glycerol tolerant (Figure 3A), whereas *c564*-GAL4; *dGyk*-IR and *c564*-GAL4; *dGK*-IR progeny on a glycerol-only diet died at rates similar to starvation (Figure 3B and 3C). When placed on a sucrose only food source, *c564*-GAL4; *dGyk*-IR and *c564*-GAL4; *dGK*-IR flies had a lifespan similar to that of control flies. Intriguingly, *c564*-GAL4; *dGyk*-IR and *c564*-GAL4; *dGK*-IR flies when placed on glycerol+sucrose mixed media also died rapidly but at a slower rate compared to glycerol alone. Due to the hygroscopic nature of glycerol, we predict hypersensitivity to food supplemented with glycerol is mainly caused by increased susceptibility to desiccation but could in part be due to an inability to metabolize glycerol (see discussion).

To test whether the glycerol hypersensitivity could be due to defective osmoregulation, we performed survivorship assays on a high salt diet (Figure S3). Both *c564*-GAL4; *dGyk*-IR and *c564*-GAL4; *dGK*-IR adult male flies were found to have a small but significant decrease in survivorship on a high salt diet (3.5% and 4.0%) compared to controls.

### Identification of a glycerol hypersensitive transposon insertion *dGyk* hypomorph

**Figure 4:**
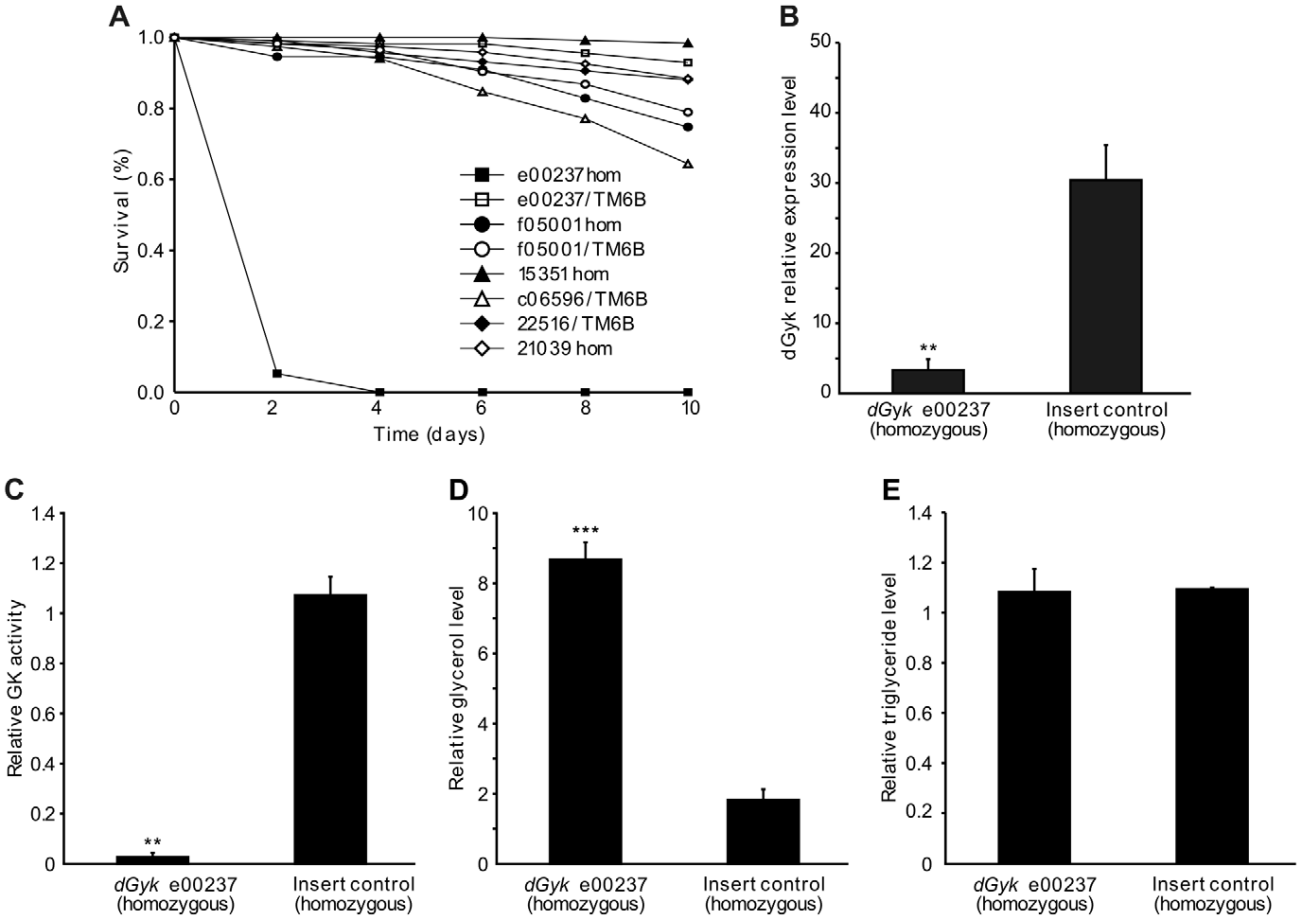
Identification of transposon insertion *dGyk* hypomorph. Using glycerol hypersensitivity as a screen-able phenotype, we tested 6 fly lines with transposon insertions that map to the genomic loci for *dGyk* (e00237, 21039, and 22516) and *dGK* (f05001, 15351, and c06596). For each line, survival assays were performed using 7–10 day old male flies by placing the flies (n>100) on glycerol (1 M) and 1.3% agarose as food source. One fly line (*dGyk* ^e00237^ homozygous) was found to be glycerol hypersensitive (A). Heterozygous *dGyk* ^e00237^/TM6B flies were not glycerol hypersensitive. The 22516 and c06596 fly lines were homozygous lethal *i.e.* homozygous flies could not be assayed. Analysis of *dGyk* ^e00237^ homozygous 3^rd^ instar larvae revealed decreased *dGyk* expression (B), decreased GK activity (C), elevated glycerol (D), and normal triglyceride levels (E). For a control fly line, a transposon insertion line was used that had an identical type of P element (pBACRB) as used to create the *dGyk* ^e00237^ fly line. Survival analysis was performed using the log-rank test on the Kaplan and Meier data. Otherwise statistical analysis was performed using the Student's *t*-test. **P<0.01, ***P<0.001.

To provide additional evidence for a role of *dGyk* and *dGK* in glycerol hypersensitivity, we screened fly stocks with transposon insertions that mapped to *dGyk* (e00237, 22516, and 21039) or *dGK* (f05001, 15351, c06596) by placing the fly lines on a glycerol-only diet (Figure 4A). This identified one glycerol hypersensitive homozygous *piggyBac* transposable element insertion (*dGyk* ^e00237^). Further characterization of this fly stock revealed decreased *dGyk* expression, decreased GK activity, elevated glycerol levels, and normal triglyceride levels (Figures 4B–E). Although *dGyk* ^e00237^ homozygous flies were fertile, fly cultures failed to thrive. Flanking sequence of the P element insertion for *dGyk* ^e00237^ (GenBank id. CZ478131) reveals the insertion site to be located 50 bp upstream of the splice acceptor site within intron 1. It is likely that this insertion disrupts the branch point consensus sequence resulting in reduced splicing efficiency.

### Suppression of glycerol hypersensitivity using *dGyk* and *dGK* transgenes

In order to perform phenotype rescue experiments, we first created stable and viable RNAi knockdown lines by placing *c564*-GAL4 and the RNAi construct on chromosome 2 and 3 respectively, over a chromosome 2+3 translocated balancer, t(2;3)SM6;TM6B (see methods for chromosome balancing information). Therefore, the GAL4 driver and RNAi construct co-segregate during crosses. The genotypes were: *c564*-GAL4; *dGyk*-IR/t(2;3)SM6;TM6B and *c564*-GAL4; *dGK*-IR/t(2;3)SM6;TM6B. Using a glycerol+sucrose food source we found rate of death correlated with glycerol concentration *i.e.* higher glycerol concentration resulted in a faster rate of death (Figure S4). Also, we observed that male RNAi knockdown flies are more glycerol hypersensitive than females (Figure S5). Therefore for our survivorship assays, males were separated from females to avoid distortion of the survival curves. Glycerol concentrations were optimized for survivorship assays to be performed over 10 days: 1.5 M and 2 M glycerol for *c564*-GAL4; *dGyk*-IR males and females, respectively; 3.0 M glycerol for *c564*-GAL4; *dGK*-IR males and females.

**Figure 5:**
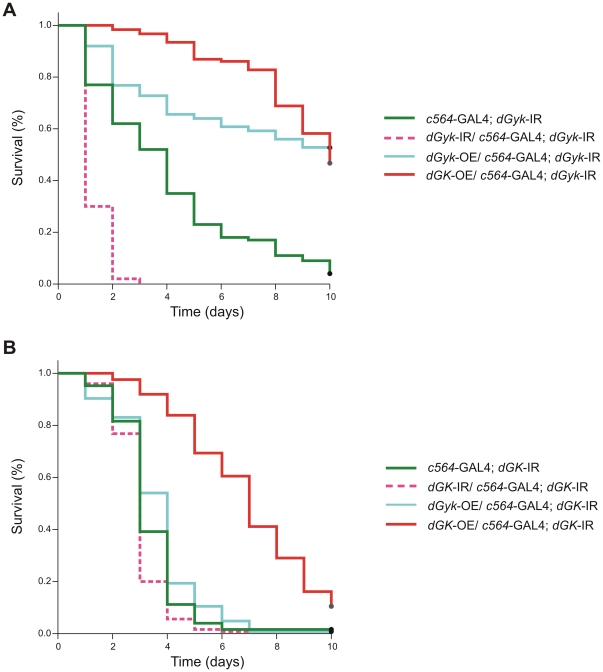
Transgenic suppression of glycerol hypersensitivity. A) Both over-expression constructs *dGyk*-OE and *dGK*-OE suppressed glycerol hypersensitivity of *c564*-GAL4; *dGyk*-IR flies. Additionally, enhanced glycerol hypersensitivity was observed for *dGyk*-IR/*c564*-GAL4; *dGyk*-IR flies. B) Suppression of glycerol hypersensitivity was observed for *dGK*-OE/*c564*-GAL4; *dGK*-IR flies but not dGyk-OE/*c564*-GAL4; *dGK*-IR flies. *dGK*-IR/*c564*-GAL4; *dGK*-IR flies did not show significantly enhanced glycerol hypersensitivity. For each genotype, n>100. Survivorship curves were analyzed using a Log-rank test on the Kaplan and Meier data.

Using the *c564*-GAL4; *dGyk*-IR/t(2;3)SM6;TM6B and *c564*-GAL4; *dGK*-IR/t(2;3)SM6;TM6B stable knockdown fly lines, we performed rescue of phenotype experiments by crossing to *dGyk*-OE or *dGK*-OE fly lines (Figure 5). Additionally, we investigated the effect of 2 copies of *dGyk*-IR or 2 copies of *dGK*-IR on glycerol hypersensitivity. Interestingly, *c564*-GAL4; *dGyk*-IR glycerol hypersensitivity was suppressed using either *dGyk*-OE or *dGK*-OE. For *c564*-GAL4; *dGK*-IR flies, glycerol hypersensitivity was suppressed using *dGK*-OE but not by *dGyk*-OE. We also found *c564*-GAL4/*dGyk*-IR; *dGyk*-IR flies were more glycerol hypersensitive than *c564*-GAL4; *dGyk*-IR flies. Glycerol hypersensitivity was not significantly enhanced in *c564*-GAL4/*dGK*-IR; *dGK*-IR flies compared to *c564*-GAL4/*dGK*-IR flies.

### A genetic modifier screen utilizing glycerol hypersensitivity phenotype

**Figure 6:**
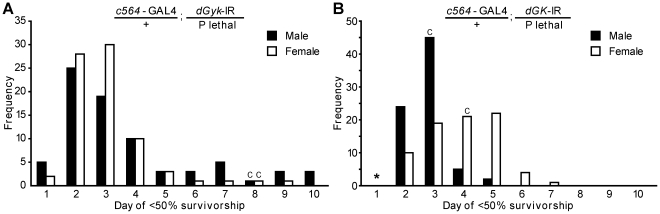
Pilot modifier screen performed using glycerol hypersensitivity phenotype. We screened 77 lethal transposon insertion mutant fly lines by crossing to (A) *c564-*GAL4; *dGyk*-IR/t(2;3)SM6;TM6B or (B) *c564*-GAL4; *dGK*-IR/t(2;3)SM6;TM6B fly lines (see methods for breeding strategy). Groups of male or female progeny (n = 20–25) with the genotypes *c564*-GAL4; *dGyk*-IR/P element or *c564*-GAL4; *dGK*-IR/P element were collected and placed on an optimized food source consisting of glycerol and sucrose (see methods for assay details). Survivorship was assayed for 10 days and plots created for day of <50% survivorship versus frequency. * one synthetic lethal cross identified. “C” indicates day of <50% survivorship for control flies, genotypes *w^1118^*; *c564*-GAL4; *dGyk*-IR or *w^1118^*; *c564*-GAL4; *dGK*-IR.

To test whether our glycerol hypersensitive survival assay could detect genetic modifier loci, we crossed 77 lethal transposon insertion mutants to the stable *c564*-GAL4; *dGyk*-IR and *c564*-GAL4; *dGK*-IR fly lines (as described in methods). All lethal transposon insertion mutants mapped to chromosome 3 and contained the *rosy* eye color marker on a *rosy* null background (see Table S2 for genotypes) and offspring of interest separated based on absence of balancer chromosome markers e.g. RNAi construct/+; GAL4 driver/P element. Male and female flies were separated and survivorship assays performed on the optimized glycerol+sucrose diet. The day of <50% survival was noted for progeny from each cross and results plotted (Figure 6).

From the 50% survival plots, top enhancers and suppressors of glycerol hypersensitivity were identified. For (*dGyk*-IR; *c564*-GAL4)/P element flies, this totaled ∼14% of lethal transposon insertion mutants tested. For (*dGK*-IR; *c564*-GAL4)/P element flies, this totaled ∼4% of lethal transposon insertion mutants tested.

One synthetic lethal cross was identified (*c564*-GAL4/+; *dGK*-IR/P element) that mapped to the gene encoding Na^+^-K^+^ ATPase alpha subunit. Two mutations are synthetically lethal if flies with either of the single mutations are viable but flies with both mutations are inviable. In this case, both RNAi flies and the heterozygous lethal transposon insertion mutant flies were viable but a combination of *c564*-GAL4/+; *dGK*-IR and heterozygous lethal transposon insertion was inviable. Originally identified in a double P element insertion, synthetic lethality was confirmed in a second lethal transposon insertion mutant mapping to the *Na^+^-K^+^ ATPase alpha subunit* gene. Further investigation of the cause of the synthetic lethality is required to confirm *Na^+^-K^+^ ATPase alpha* as a modifier of glycerol hypersensitivity.

Strikingly, the majority of *c564*-GAL4; *dGyk*-IR/P element flies were more glycerol hypersensitive than the control *w^1118^*; *c564*-GAL4; *dGyk*-IR flies (Figure 6A). A similar but weaker trend was observed for of *c564*-GAL4; *dGK*-IR/P element flies (Figure 6B). We suspected that the *rosy* null genetic background of the lethal transposon insertion mutants might be the cause of the enhanced glycerol hypersensitivity.

### Glycerol hypersensitivity is affected by eye pigmentation null mutations

**Figure 7:**
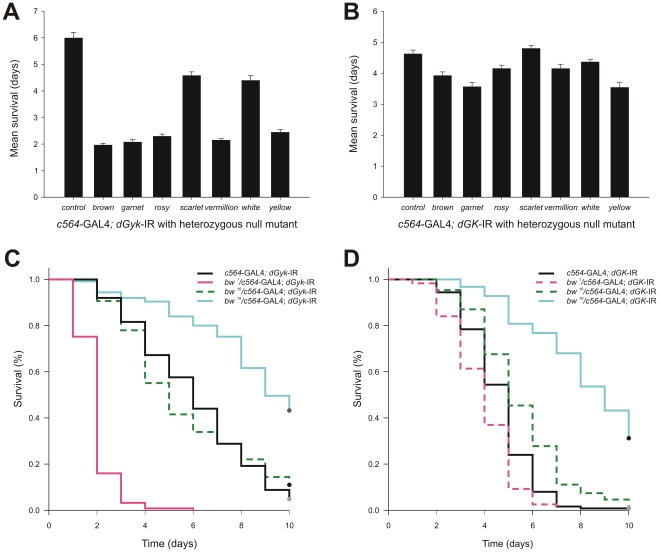
Glycerol hypersensitivity is affected by eye pigmentation null mutations. Flies heterozygous for eye pigmentation null mutations in *trans* to c564-GAL4 driver and RNAi construct were found to show enhanced glycerol hypersensitivity. Mean survival times are shown for A) *dGyk*-IR progeny and B) *dGK*-IR progeny. RNAi progeny from Canton-S flies were used as wild type. Progeny from *yellow* mutant flies were included as a control for desiccation sensitive flies. Additional glycerol hypersensitive survival assays were performed using flies heterozygous for 3 *brown* mutations (*bw^1^, bw^16^,* and *bw^19^*) in *trans* to C) *c564*-GAL4; *dGyk*-IR and D) *c564*-GAL4; *dGK*-IR. These results show that glycerol hypersensitivity can be either enhanced (*bw* ^1^) or suppressed (*bw* ^19^), suggesting the *brown* locus to be an important genetic modifier of glycerol hypersensitivity. Glycerol hypersensitive survivorship assays used 6–10 day old female flies, n>100 (see methods for assay conditions). Survivorship curves were analyzed using a Log-rank test on the Kaplan and Meier data. Control data is shown in Figures S6 and S7.

Results from the preliminary modifier screen indicated that the *rosy* null background of the lethal transposon insertion mutant flies might affect glycerol hypersensitivity. To investigate whether this effect was *rosy* specific or a feature of eye color mutants, we tested a panel of eye color mutants by performing glycerol hypersensitivity survivorship analysis on *c564*-GAL4; *dGyk*-IR and *c564*-GAL4; *dGK*-IR flies that also possessed a heterozygous null mutation in an eye pigmentation gene. Additionally, we included yellow fly mutants; these flies have yellow body cuticles and have previously been shown to be sensitive to desiccation [31]. This revealed that the mutants *brown*, *garnet*, *rosy*, *vermillion*, and yellow, all enhanced glycerol hypersensitivity (Figures 7A and 7B). These flies were all tolerant over 10 days to a sucrose only diet (Figures S6A and S6B). Although control flies (heterozygous pigmentation null mutation in *trans* to the *c564*-GAL4 driver) were tolerant over 10 days for sucrose only and glycerol+sucrose food sources, yellow flies did show some glycerol hypersensitivity after 10 days (Figures S6C and S6D).

Further screening of *brown* mutants resulted in a variety of outcomes (Figures 7C and 7D): a DNA insertion null mutant enhanced glycerol hypersensitivity (*bw^1^*); a premature stop codon mutant (*bw^19^*) suppressed glycerol hypersensitivity; a brown mis-sense mutation (*bw^16^*) did not significantly alter glycerol hypersensitivity compared to controls. These results suggest the *brown* gene to be an important genetic modifier locus of both the *dGyk* and *dGK* glycerol hypersensitive phenotype.

To test whether eye pigmentation homozygous null mutants were themselves glycerol hypersensitive we performed survivorship assays on glycerol+sucrose and sucrose only food sources (Figure S7). Again we included the yellow mutant fly line as a positive control. This revealed a large variation in glycerol hypersensitivity with several homozygous null mutants showing significantly enhanced glycerol hypersensitivity for example the *garnet* (*g^1^*) homozygous null mutation. Interestingly the yellow mutant fly line was both glycerol hypersensitive and sucrose hypersensitive. These results reinforce the important role that *dGyk* and *dGK* in addition to *brown*, *garnet*, *rosy*, *vermillion* and *yellow* play in glycerol hypersensitivity.

## Discussion

The conservation of metabolic and signaling pathways between *Drosophila* and mammals makes it an excellent model organism to study human disease genes (reviewed in [32]). Additionally, *Drosophila* has recently emerged as an important organism for the study of lipid biology [33] and genes involved in regulation of metabolism [34]. Here we have used *Drosophila* as a model organism for the study of the human metabolic disorder glycerol kinase deficiency (GKD). The presence in *Drosophila* genome of all the genes encoding enzymes involved in glycerol metabolism in humans [19] makes *Drosophila* a relevant model organism for the study of glycerol metabolism. However, it should be noted that there are some important differences between insect and mammalian fat metabolism. While both mammalian and insect systems use lipoproteins for lipid transport, the major lipid transported in insects is diacylglycerol whereas in mammals it is triacylglycerol [35], [36]. Nevertheless, a genetically tractable *Drosophila* model for GKD would be a powerful tool for the study of GKD.

Glycerol kinase phosphorylates glycerol to glycerol 3-phosphate in an ATP-dependent reaction. Therefore reduced GK activity should cause elevated levels of glycerol. As expected, RNAi targeting of *dGyk* and *dGK* expression resulted in knockdown flies with reduced *dGyk* and *dGK* RNA expression, reduced GK activity, and elevated glycerol levels. These are similar characteristics to human GKD patients with hyperglycerolemia and indicate that we have successfully made a *Drosophila* model for GKD. Interestingly, individual knockdown of *dGyk* or *dGK* was sufficient to reduce GK phosphorylation indicating that both are required to maintain normal glycerol levels.

Further characterization of RNAi progeny identified a glycerol hypersensitive phenotype whereby flies would rapidly die when placed on a food source supplemented with glycerol. Identification of a glycerol hypersensitive *piggyBac* transposable insertion *dGyk* hypomorph confirmed glycerol hypersensitivity to be an authentic phenotype due to reduced glycerol kinase activity. However, without performing a precise excision and reversion of the phenotype there is a small possibility that the glycerol hypersensitivity could be due to some other linked recessive mutation in the homozygous *dGyk* ^e00237^ fly line.

Although glycerol hypersensitivity could in part be due to an inability to metabolize glycerol, knockdown flies also died rapidly when placed on complete fly food supplemented with glycerol indicating toxicity to glycerol. Due to the hygroscopic nature of glycerol, we suspect glycerol hypersensitivity is a desiccation sensitive phenotype and suggests a novel role for glycerol kinase in desiccation resistance. Additionally, the control of glycerol levels in insects such as the goldenrod gall fly, *Eurosta solidaginis* [27] is known to play an important role in desiccation tolerance. Therefore we predict glycerol hypersensitivity is due to a combination of altered glycerol levels in the glycerol kinase RNAi knockdown flies in addition to the hygroscopic nature of glycerol in the fly food. Interestingly, males were more glycerol hypersensitive than female *Drosophila*. One possible explanation for this difference is that females are larger than the males and contain more water leading to suppression of glycerol hypersensitivity.

Indirect evidence supporting glycerol hypersensitivity as a desiccation tolerance phenotype was obtained by the finding that *yellow* homozygous null mutant flies, previously shown to be desiccation sensitive using a starvation/desiccation assay [31] were also glycerol hypersensitive (Figure S7). It should be noted that the function of the yellow protein, which is known to play a role in black melanin synthesis in the body cuticle [37], has not been fully elucidated.

As mentioned previously, human glycerol kinase expression is highest in the liver [13]. Therefore, we used the *c564*-GAL4 driver which has previously been shown to drive expression of GAL4 in the larval fat body [29], a tissue that plays an important role in energy metabolism similar to that of mammalian liver [30]. The *c564*-GAL4 driver has previously been used to drive RNAi expression in adult flies to explore gene function in relation to fat metabolism [34]. However, it should be noted that in adult flies, the GAL4 expression pattern driven by *c564*-GAL4 is not fat body specific. Using a GFP reporter construct, GFP expression was observed to have a much wider expression pattern that included fat body, gut, malpighian tubules, salivary glands and eye. Therefore we speculate that glycerol hypersensitivity might not be due to decreased expression in the fat body alone. In addition to liver, mammalian glycerol kinase is also highly expressed in the kidney so the malpighian tubules, which perform a similar function to mammalian kidney, could be an important tissue for the glycerol hypersensitivity phenotype. Further RNAi experiments using additional GAL4 drivers might clarify which cell type/tissue is important for glycerol hypersensitivity.

One advantage of using *Drosophila* as a model organism is the ability to perform genetic modifier screens [38]. To this end, we used the glycerol hypersensitive phenotype to perform a preliminary screen of lethal transposon insertion mutants. Our aim was to show that our GKD *Drosophila* model could be used to identify genetic modifier loci. Conveniently, results of survivorship assays can be quantitatively analyzed, allowing lethal transposon insertion mutants to be ranked based on day of <50% survival and allows both suppressors and enhancers of glycerol hypersensitivity to be identified. The power of this type of screen increases with the number of lethal transposon insertion mutants screened and a full screen would be required to identify the best targets.

Using an identical set of lethal transposon insertion mutants, data analysis of the preliminary glycerol hypersensitive survivorship screen revealed a much wider distribution of 50% survival times for *dGyk*-RNAi progeny compared to *dGK*-RNAi progeny (Figure 6). This difference indicates that *dGyk* and *dGK* are likely to have some redundancy in their enzymatic activity but in addition they are likely to have some different functional roles. This is similar to the mammalian glycerol kinase which has the enzymatic activity as well as the alternative protein functions. It will be interesting to examine these different roles of the two enzymes in future studies including tissue specific expression, temporal expression, and subcellular localization.

As mentioned previously, a complete screen of available lethal transposon insertion mutants would be required to identify the best enhancers and suppressors of glycerol hypersensitivity. One candidate gene for further investigation was identified as a synthetic lethal cross that mapped to the gene encoding the ATPase alpha subunit. The ATPase is a Na^+^-K^+^ exchange pump and has been implicated in a number of cellular processes in addition to ion transport [39]–[42]. This suggests ion transport is an important cellular process that is required to maintain viability when *dGK* levels are reduced.

It was also noticed that the majority of *c564*-GAL4; *dGyk*-IR; P element progeny were more glycerol hypersensitive compared to control flies, suggesting that the *rosy* null background affects glycerol hypersensitivity. Screening of a panel of eye pigmentation null mutants (with the null mutation in trans to *c564*-GAL4; *dGyk*-IR) revealed that in addition to *rosy* mutants, the eye color mutants *brown*, *garnet*, and *vermillion* strongly enhanced glycerol hypersensitivity (Figure 7A). A similar but reduced glycerol hypersensitive enhancing effect of eye pigmentation null mutants was on *c564*-GAL4; *dGK*-IR progeny was also observed. This effect was least in female progeny. Consequently, to minimize eye color genetic background effect on glycerol hypersensitivity, future screening of lethal transposon insertion mutants will focus on *c564*-GAL4; *dGK*-IR female progeny.

Whereas the *bw^1^* null mutation of the *brown* gene resulted in strong enhancement of glycerol hypersensitivity, the *bw* ^19^ mutation resulted in suppression of glycerol hypersensitivity. Unlike the *bw^1^* null mutation, which is an insertion of DNA into the transcription unit, the *bw* ^19^ mutation is a nonsense substitution in codon 102 resulting in a premature stop codon. One explanation for this result could be that the stop codon induces exon skipping, resulting in an alternative protein that has a protective effect against glycerol hypersensitivity. Another *brown* mutant, the *bw^16^* missense mutation A78V did not significantly affect glycerol hypersensitivity, indicating this amino acid change does not alter the function of the brown protein with respect to its role in glycerol hypersensitivity. These results suggest the *brown* gene could be an important genetic modifier of the glycerol kinase RNAi glycerol hypersensitivity phenotype.

In *Drosophila* eye, pigmentation genes encode proteins with a variety of roles, for example: metabolic enzymes such as xanthine dehydrogenase (*rosy*; [43]), tryptophan 2,3-dioxygenase (*vermillion*; [44]); ATP-binding cassette (ABC) co-transporters (*white*, *brown*, *scarlet*; [45], [46]); a subunit of the AP-3 complex involved in endocytosis (*garnet*; [47]). These proteins all either modify or transport molecules of pigment precursors to pigment granules. Interestingly, an interaction between eye pigmentation genes and tau-induced neurodegeneration has recently been established in the *Drosophila* eye [48]. However, these genes are widely expressed but their non-eye roles are not understood. Our glycerol hypersensitive phenotype indicates a new role for eye pigmentation genes outside of the eye.

The ABC co-transporters white and brown act as a dimer to transport guanine-derived drosopterin precursors whereas white and scarlet transport tryptophan-derived xanthommatin precursors [49]–[52]. For *dGyk*- and *dGK*-RNAi knockdown flies, the *white* and *scarlet* mutations had a relatively small effect on glycerol hypersensitivity compared to the *brown* mutation. As both the RNAi construct and the *c564*-GAL4 driver possess a mini-white cDNA sequence, this could explain why the white mutant resulted in only a small enhancement of glycerol hypersensitivity. Therefore it is possible that white and brown dimers play a more important role in the transport of molecules in response to desiccation than white and scarlet dimers.

There are a number of other eye pigmentation mutants characterized by the fly community that could potentially also be glycerol hypersensitive. However the exact size of this group of glycerol hypersensitive mutants remains unknown. Whether these proteins all function in the same desiccation response pathway and how glycerol kinase fits into this pathway remains to be elucidated.

To determine the significance of these results in relation to glycerol kinase deficiency in humans will require further research in mammalian systems. We hypothesize that genetic variation in the human homologues of *Drosophila* eye pigmentation genes could play an important role in the phenotypic variation observed in human GKD patients. Mutations in human homologues of the white ABC transporter family cause sitosterolemia and it has been suggested that heterozygous variants in *ABC* gene mutations are implicated in several complex disorders [53].

Mutations at the *GK* (Xp21) locus cause GKD in humans. However, much remains to be understood about the underlying pathogenic mechanism and why such a wide range of phenotype severity is observed. Additionally, a role for GK alternative functions and modifier loci has still to be fully explored. Using our glycerol hypersensitive *Drosophila* model for GKD, we have found evidence showing an important role for eye pigmentation genes in determining phenotype severity. Future work will expand the glycerol hypersensitive modifier screen with the aim of identifying novel modifiers and confirm whether they are conserved between insects and mammalian systems. We conclude that RNAi targeting of *dGyk* and *dGK* in *Drosophila* is a valid model for the study of GKD and has the potential to identify genetic modifier loci that could help unravel the complexity of the pathogenic mechanism observed in GKD patients.

## Materials and Methods

### Constructs and *Drosophila* stocks

For all RNAi and over-expression constructs, cDNA fragments were PCR amplified from Berkeley *Drosophila* Genome Project cDNA clones GH12641 and GH18680 that contain complete coding regions for *dGyk* and *dGK* respectively. For RNAi constructs, PCR amplified cDNAs were initially subcloned into the *pHIBS* vector before further subcloning as an inverted repeat (IR) into the *pUDsGFP* vector [28]. The *pUDsGFP* construct co-expresses GFP with the inverted repeat, allowing easy recognition of GFP-positive larvae that possess both the RNAi construct and the GAL4 driver. Primers pairs for PCR amplification were as follows: dGyk-IR-for d5′-AGTTGGATCCGAAATAATCACGATTGGAA-3′ and dGyk-IR-rev d5′- AGTTGGTACCTAGTAATCCGTGCGTTGAG-3′; dGK-IR-for d5′- AGTTGGATCCCTGCTCAAGACGTTCGGTA-3′ and dGK-IR-rev d5′- AGTTGGTACCTCGAACTGGCAGAGATTGA-3′. For over-expression constructs, the complete coding regions for *dGyk* and *dGK* were PCR amplified and subcloned into the *pEX-UAS* vector [54]. Primers for PCR amplifying the complete coding regions for *dGyk* and *dGK* were as follows: dGyk-for d5′- ATTGCGGCCGCAAAAAAAATGGATTCTCCC-3′ and dGyk-rev d5′- ATTTCTAGATGATCACGCTCCGTCAAAGGC-3′; dGK-for d5′- ATTGCGGCCGCAAGCAGCATGACCGAGGGC-3′ and dGK-rev d5′- AGCTCTAGATATTTACTGGCCACTCGCAGC -3′. Microinjection of DNA constructs, identification of transformants and balancing was performed by BestGene Inc (Chino Hills, CA).

Stable knockdown lines containing both GAL4 driver (*c564*-GAL4 on chromosome 2) and RNAi construct (on chromosome 3) were generated by standard genetic crosses using appropriate balancer chromosomes and maintained over a translocated chromosome 2–3 balancer - t(2;3)SM6;TM6B, from the Bloomington *Drosophila* stock center (BDSC). Balancer chromosomes contain nested chromosomal inversions that disrupt crossing over [55]. They also contain marker mutations that are often recessive lethals themselves. Therefore, stable heterozygous stocks for transgenic constructs or mutations can be used for crosses and the genotype of progeny reliably inferred by presence/absence of the balancer chromosome marker.

All GAL4 driver fly stocks were obtained from the BDSC: *P{TubP-GAL4}* [56]; *P{GawB}c564* [29]; *P{GawB}how[24B]* [23]; *P{GawB}Elav[C155]* [57]; *P{GMR-GAL4}* [58]. For P insertions mapping to *dGyk* and *dGK*, stocks 15351, 21039, 22516 were obtained from the BDSC and the stocks c06596, e00237, and f05001 were obtained from the Exelixis collection at Harvard medical school. Bloomington stock 17932 was used as a control fly line for e00237. Genotypes of all lethal transposon insertion mutants stocks are listed in Table S2.

Eye pigmentation mutant flies were originally obtained from BDSC: *brown* (*bw^1^*, *bw^16^*, *bw^19^*), *garnet* (*g^1^*), *rosy* (*ry^1^*), *scarlet* (*st^1^*), *vermillion* (*v^1^*), and *yellow* (*y^1^*). The *bw^16^* and *bw^19^* mutants were originally created and characterized by Kondrashov, A. *et al*., unpublished. The Canton-S (wild type control) and *w^1118^* flies were a kind gift from the laboratory of Dr G. Jackson.

### RNA preparation and quantitative real-time PCR

RNA was extracted from ten 3^rd^ instar larvae using the RNAeasy® mini kit (Invitrogen, Carlsbad, CA) according to manufacturer's instructions. Total RNA (1 µg) was used for first-strand cDNA synthesis using the SuperScript® III reverse transcriptase and random primers (Invitrogen). Quantitative real-time PCR (qRT-PCR) was performed using PerfeCTa™ SYBR® Green FastMix™ ROX (Quanta Biosciences, Gaithersburg, MD) on a StepOne™ real time PCR machine (Applied Biosystems, Foster City, CA). Fold differences for each of the genes tested were calculated using the 2[Delta][Delta]CT method [59]. All reactions were performed in triplicate. Expression levels of *dGyk* and *dGK* were normalized to *RpII*. Primers were designed using Primer3 software [60] and synthesized by Integrated DNA Technologies (San Diego, CA). Primer sequences were as follows: *dGyk* d5′TAGGCATAACATCGGTTCTGG3′ and d5′GCCTTCCGTCCTAGTTGGTAG-3′; *dGK* d5′AGACGACAATCGTCTGGGATG3′ and d5′CACGATCTGCTCCACTGTAG3′; *RpII* d5′AAGGCTATGGTGGTGTCTGG3′ and d5′GCTTACCCTCCACGTTCTGT3′.

### Glycerol kinase activity assay

Glycerol kinase activity was determined by using a radiolabeled assay as previously reported [61]. Briefly, protein was extracted in homogenization buffer (1% KCl; 1 mM EDTA+Complete protease inhibitor (Roche, Indianapolis, IN)) from two groups of three 3^rd^ instar larvae and assayed in duplicate using 4 µg of total cellular protein for 20 min, assay conditions and reaction mix previously determined to be optimal for 3^rd^ instar larvae protein extracts (data not shown). Incorporation of ^14^C-glycerol (GE Healthcare, Piscataway, NJ) into glycerol 3-phosphate was measured using a scintillation counter and GK activity of test samples calculated by comparison to a standard curve.

### Glycerol and triglyceride assays

For glycerol and triglyceride measurements, batches of three 3^rd^ instar larvae were homogenized in 250 µl homogenization buffer (10 mM Tris-HCl pH 7.4, 10 mM NaCl, 1 mM EDTA, 0.5% Triton X-100) including Complete protease inhibitor (Roche). Next, 14 µl of 20% triton X-100 was added to 186 µl of the sample. After heating at 70°C (5 mins) to inactivate endogenous enzymes, samples were centrifuged for at 13000 rpm (5 mins) and the supernatant transferred to a new tube (after homogenizing the white lipid ring with the tip of the pipette). Glycerol levels were measured using Free Glycerol Reagent (Sigma-Aldrich). Triglyceride levels were determined using the L-type Triglyceride determination kit M (Wako, Richmond, VA). Results from this assay are not affected by free glycerol because all free glycerol is decomposed in an initial experimental step before the enzymatic hydrolysis of triglyceride. Values were normalized against protein concentration using the Micro BCA™ Protein Assay Kit (Thermo Scientific, Rockford, IL) and experiments were performed in triplicate for each genotype.

### Glycerol hypersensitivity survivorship assay

For each genotype, 5 batches of 20–25 flies (7-day old males) were transferred to vials containing defined food sources and incubated at 25°C. Food sources used were: starvation (1.3% agarose only), glycerol only (1 M glycerol+1.3% agarose), sucrose only (5% sucrose+1.3% agarose), glycerol+sucrose (1 M glycerol+5% sucrose+1.3% agarose). Dead flies were counted every 24 hr for survival rate calculations. Data are the average with SEM from at least 5 vials for each genotype. The mean and SEM of data was plotted and survivorship curves analyzed using a Log-rank test on the Kaplan and Meier data.

### Preliminary modifier screen

Genotypes used for screen were (*c564*-GAL4; *dGyk*-IR)/t(2;3)SM6;TM6B and (*c564*-GAL4; *dGK*-IR)/t(2;3)SM6;TM6B. Note: RNAi construct lines were different to those used in other experiments but progeny from *Tub*-GAL4 driver flies were shown to have decreased *dGyk*- or *dGK*-RNA expression, decreased GK activity and elevated glycerol. For glycerol hypersensitivity assays, food sources consisted of 5% sucrose and 1.3% agarose with the glycerol concentration optimized for survivorship assays to be performed over 10 days. For each screen, glycerol concentrations were as follows: *dGyk* male 1.5 M, *dGyk* female 2.0 M, *dGK* male 3.0 M, *dGK* female 3.0 M. Crosses were set up between RNAi knockdown males and virgin lethal transposon insertion mutants (see Table S2). Progeny were genotyped based on presence or absence balancer chromosome markers. Sex specific survivorship assays were performed by placing 7–10 day old flies (n = 20–25) on glycerol+sucrose media and dead flies counted every 24 hr. Top targets identified were ranked by repeating survivorship assays (n>100).

### Glycerol hypersensitive screen of eye color mutants

Glycerol hypersensitivity survivorship assays were performed as previously described using eye pigmentation mutant flies. As several of the genes for the color mutants are located on the X chromosome, we crossed virgin female color mutant flies to stable knockdown fly lines (*c564*-GAL4; *dGyk*-IR)/t(2;3)SM6;TM6B and (*c564*-GAL4; *dGK*-IR)/t(2;3)SM6;TM6B. Assays were performed using 8–10 day old female progeny.

### Statistical analysis

Survival curves were analyzed using a Log-rank test on the Kaplan and Meier data. One way ANOVA with post-hoc pair wise multiple comparison procedures (Tukey Test) were applied to qRT-PCR and biochemical data where stated. Student's *t*-test was used where stated and error bars represent SEM.

## Supporting Information

Figure S1 **GFP expression correlates with phenotype severity.** Western blot analysis was performed for GFP in knockdown roaming 3^rd^ instar larvae (the *pUds*GFP RNAi vector co-expresses GFP). Beta-actin was used as the control (Methods S1). Relative levels of GFP would provide an indirect measure of the inverse repeat (IR) expression levels, for example greater GFP levels would indicate greater levels IR expression and infer greater knockdown of the target gene expression levels. For *dGyk*-IR; *Tub*-GAL4 larvae, western blot analysis revealed higher GFP levels in knockdown 3^rd^ instar larvae that died before eclosion than in 3^rd^ instar larvae that developed into glycerol hypersensitive adult flies. A similar trend was observed for *dGK*-IR; *Tub*-GAL4 3^rd^ instar larvae. Therefore larval lethality is likely due to lower levels of dGyk and dGK due to greater expression of the *dGyk*-IR and *dGK*-IR construct.
(TIFF)

Figure S2 **Control RNA expression data for** **Figure 1** **.** Relative RNA expression levels of *dGyk* and *dGK* RNA were quantitated for parental fly lines used to generate RNAi knockdown flies (A and B) and over-expression flies (C and D). For each group, values were not found to be statistically different. Statistical analysis using ANOVA was performed by comparison to GAL4 fly line.
(TIFF)

Figure S3 **Adult** ***c564-*** **GAL4;** ***dGyk-*** **IR and** ***c564*** **-GAL4;** ***dGK*** **-IR are hypersensitive to NaCl compared to control flies.** Survival assays were performed using 7-day old male progeny placed on complete Jazz-mix *Drosophila* food (Fisher, Pittsburgh, PA) supplemented with 3.5% NaCl (black bars) or 4.0% NaCl (white bars). For each genotype, 5 vials of 20–25 flies were counted every 24 hr until 100% lethality. Survival analysis using the log-rank test on the Kaplan and Meier data was used to calculate mean survival time, standard error and significance. *P<0.05, **P<0.01.
(TIFF)

Figure S4 **Glycerol hypersensitive survivorship assay optimization.** Adult flies A) *c564-*GAL4; *dGyk-*IR and B) *c564*-GAL4; *dGK*-IR were placed on food sources containing glycerol (0–4 M glycerol; 5% sucrose; 1.3% agarose) and flies counted every 24 hr. Survival curves were plotted for each glycerol concentration. Each assay used 8–10 day old female flies, n = 25.
(TIFF)

Figure S5 **Glycerol hypersensitive sex differences.** For RNAi knockdown flies, males were found to be more hypersensitive to glycerol than females. Glycerol hypersensitive survivorship assays were performed using single sex groups of flies. A) *c564-*GAL4; *dGyk-*IR adult flies on 1.5 M glycerol, 5% sucrose, 1.3% agarose. B) *c564*-GAL4; *dGK*-IR adult flies on 3 M glycerol, 5% sucrose, 1.3% agarose. Each assay used 8–10 day old flies, n>100. Survivorship curves were analyzed using a Log-rank test on the Kaplan and Meier data. * P<0.05, ***<0.001.
(TIFF)

Figure S6 **Control survivorship assays.** Flies heterozygous for eye pigmentation null mutations in trans to A) *c564-*GAL4; *dGyk*-IR and B) c*564-*GAL4; *dGK*-IR are tolerant to a sucrose only food source over 10 days (5% sucrose, 1.3% agarose). C) Using a 2 M glycerol, 5% sucrose food source, heterozygous pigmentation null mutations in *trans* to the *c564-*GAL4 driver show some glycerol hypersensitivity after 10 days. D) Using a 3 M glycerol, 5% sucrose food source, heterozygous pigmentation null mutations in *trans* to the *c564-*GAL4 driver show increased glycerol hypersensitivity after 10 days compared to the 2 M glycerol 5% sucrose food source. In both C and D, control flies are more tolerant to glycerol than the *c564-*GAL4; *dGyk*-IR and c*564-*GAL4; *dGK*-IR knockdown flies (Figure 7). As a positive control, survivorship assays were performed using progeny from *yellow* flies, a mutant fly line previously shown to be desiccation sensitive. For each genotype, female flies (n>100) were aged 6–10 days on complete fly food before placing on the defined food source.
(TIFF)

Figure S7 **Survival analysis of pigmentation homozygous null mutant flies on defined food sources.** A) 3 M glycerol, 5% sucrose food source, and B) 5% sucrose only food source. As a positive control, survivorship assays were performed using progeny from *yellow* flies, a mutant fly line previously shown to be desiccation sensitive. For each genotype, female flies (n>100) were aged 6–10 days on complete fly food before placing on the defined food source. Flies were counted every 24 hr until all were dead.
(TIF)

Table S1 **Summary of RNAi, over-expression, and P element insertion fly lines.**
(DOC)

Table S2 (DOC)

Methods S1 **Western Blotting.**
(DOCX)
