# Supplementary figures and images for: Glycerol Hypersensitivity in a Drosophila Model for Glycerol Kinase Deficiency Is Affected by Mutations in Eye Pigmentation Genes
Source: PLoS One. 2012 Mar 9;7(3):e31779. doi: 10.1371/journal.pone.0031779 (PMC3302884; doi:10.1371/journal.pone.0031779)

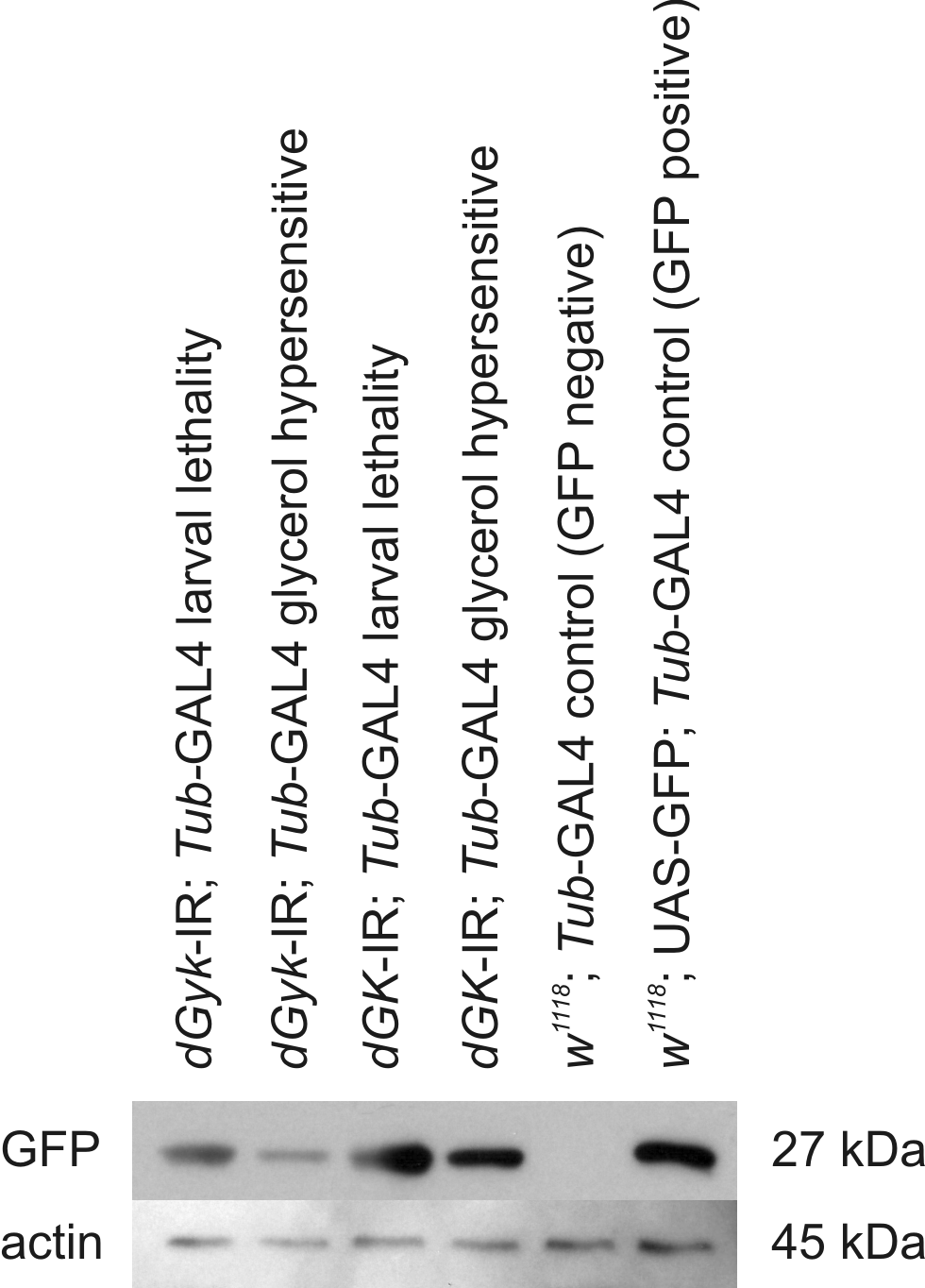

Supplement: Figure S1 — GFP expression correlates with phenotype severity. Western blot analysis was performed for GFP in knockdown roaming 3rd instar larvae (the pUdsGFP RNAi vector co-expresses GFP). Beta-actin was used as the control (Methods S1). Relative levels of GFP would provide an indirect measure of the inverse repeat (IR) expression levels, for example greater GFP levels would indicate greater levels IR expression and infer greater knockdown of the target gene expression levels. For dGyk-IR; Tub-GAL4 larvae, western blot analysis revealed higher GFP levels in knockdown 3rd instar larvae that died before eclosion than in 3rd instar larvae that developed into glycerol hypersensitive adult flies. A similar trend was observed for dGK-IR; Tub-GAL4 3rd instar larvae. Therefore larval lethality is likely due to lower levels of dGyk and dGK due to greater expression of the dGyk-IR and dGK-IR construct. (TIFF) [file pone.0031779.s001.tif]

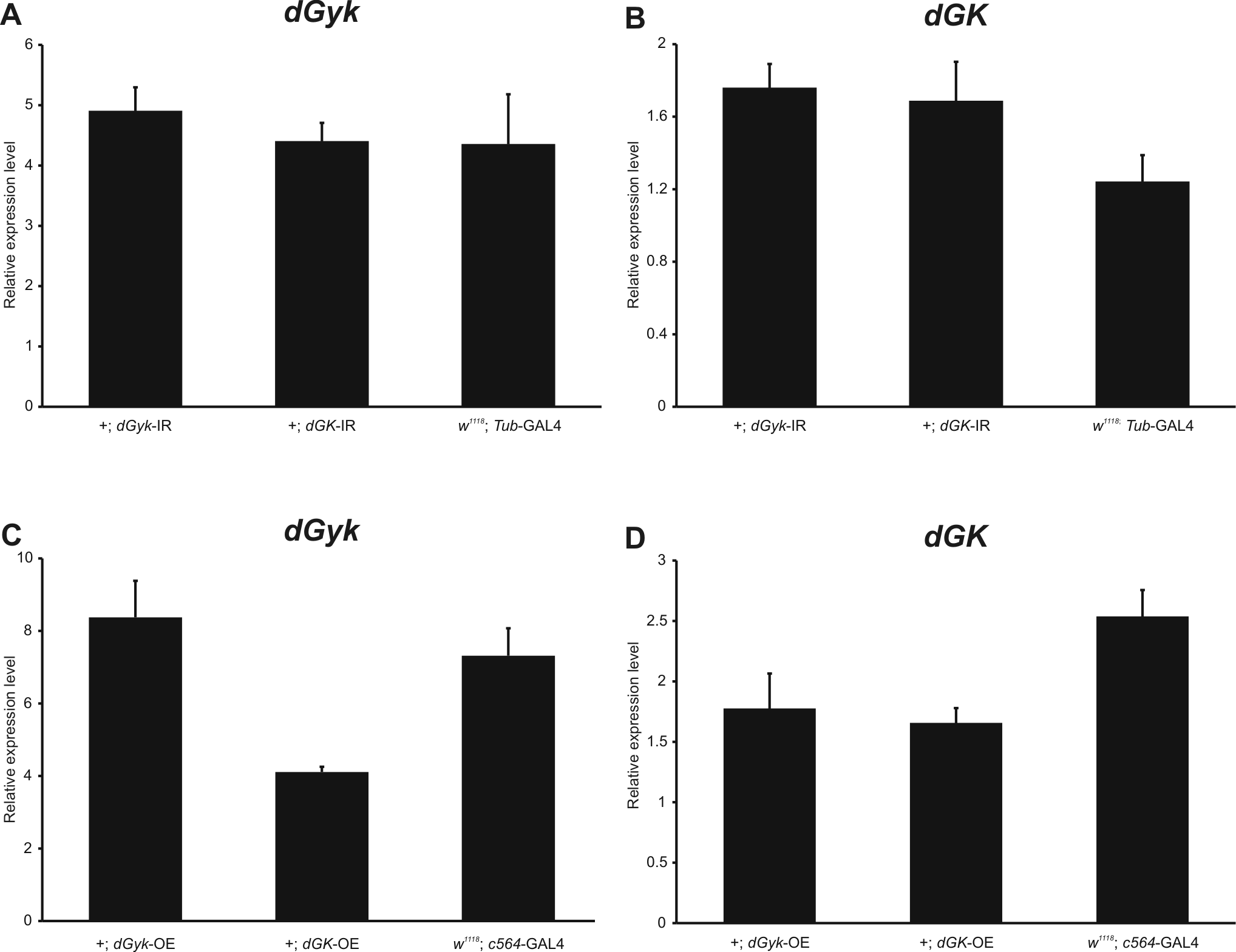

Supplement: Figure S2 — Control RNA expression data for Figure 1 . Relative RNA expression levels of dGyk and dGK RNA were quantitated for parental fly lines used to generate RNAi knockdown flies (A and B) and over-expression flies (C and D). For each group, values were not found to be statistically different. Statistical analysis using ANOVA was performed by comparison to GAL4 fly line. (TIFF) [file pone.0031779.s002.tif]

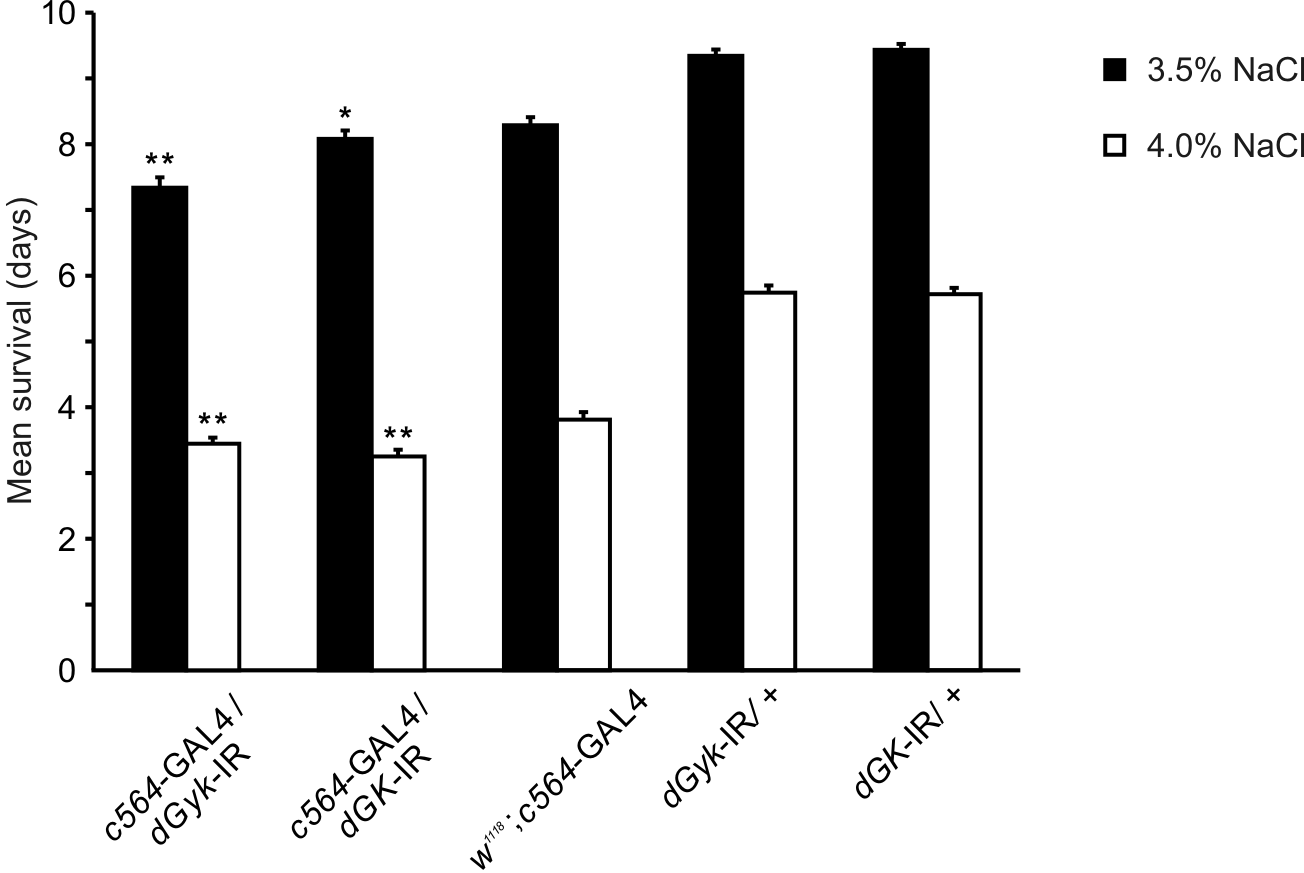

Supplement: Figure S3 — Adult c564- GAL4; dGyk- IR and c564 -GAL4; dGK -IR are hypersensitive to NaCl compared to control flies. Survival assays were performed using 7-day old male progeny placed on complete Jazz-mix Drosophila food (Fisher, Pittsburgh, PA) supplemented with 3.5% NaCl (black bars) or 4.0% NaCl (white bars). For each genotype, 5 vials of 20–25 flies were counted every 24 hr until 100% lethality. Survival analysis using the log-rank test on the Kaplan and Meier data was used to calculate mean survival time, standard error and significance. *P<0.05, **P<0.01. (TIFF) [file pone.0031779.s003.tif]

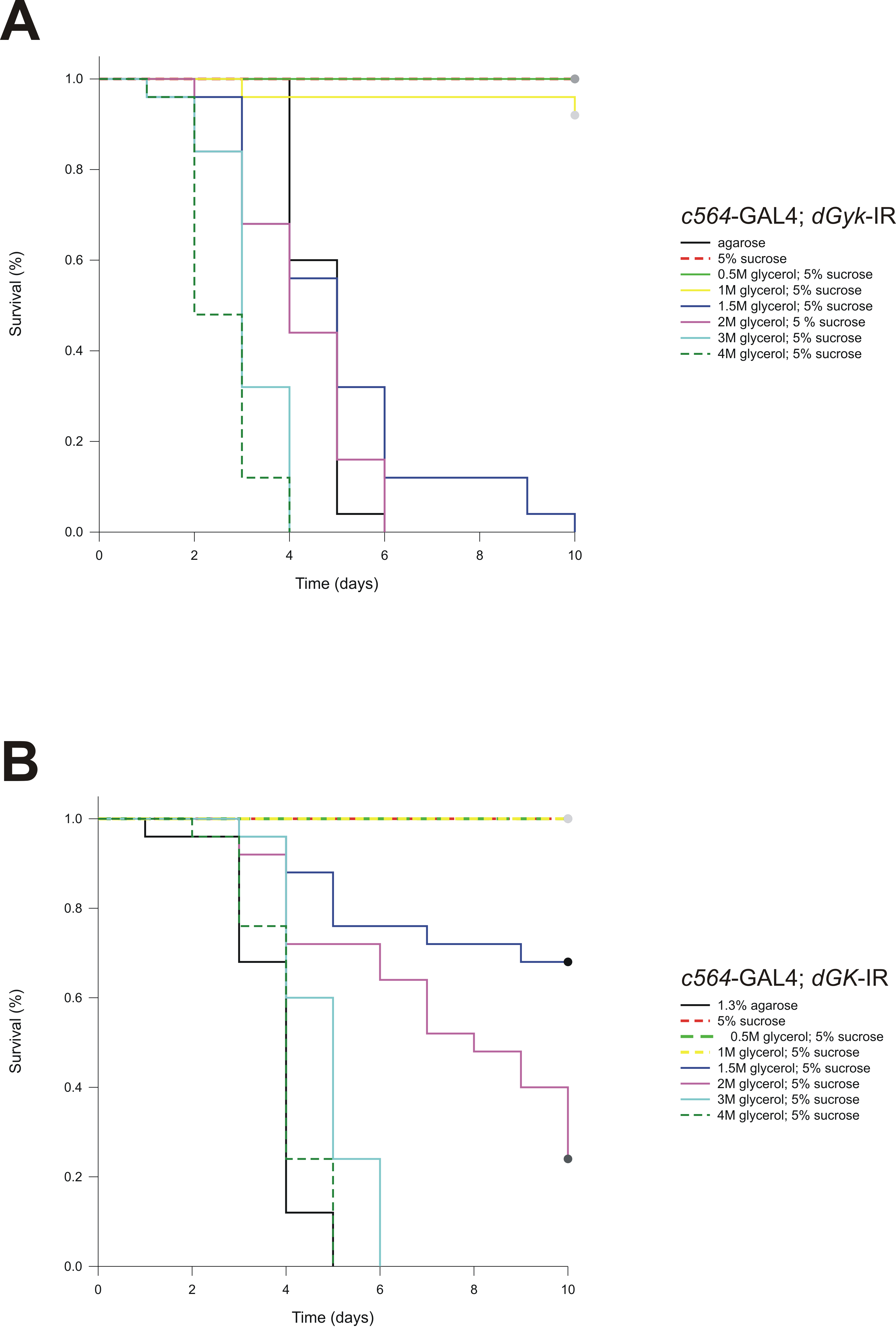

Supplement: Figure S4 — Glycerol hypersensitive survivorship assay optimization. Adult flies A) c564-GAL4; dGyk-IR and B) c564-GAL4; dGK-IR were placed on food sources containing glycerol (0–4 M glycerol; 5% sucrose; 1.3% agarose) and flies counted every 24 hr. Survival curves were plotted for each glycerol concentration. Each assay used 8–10 day old female flies, n = 25. (TIFF) [file pone.0031779.s004.tif]

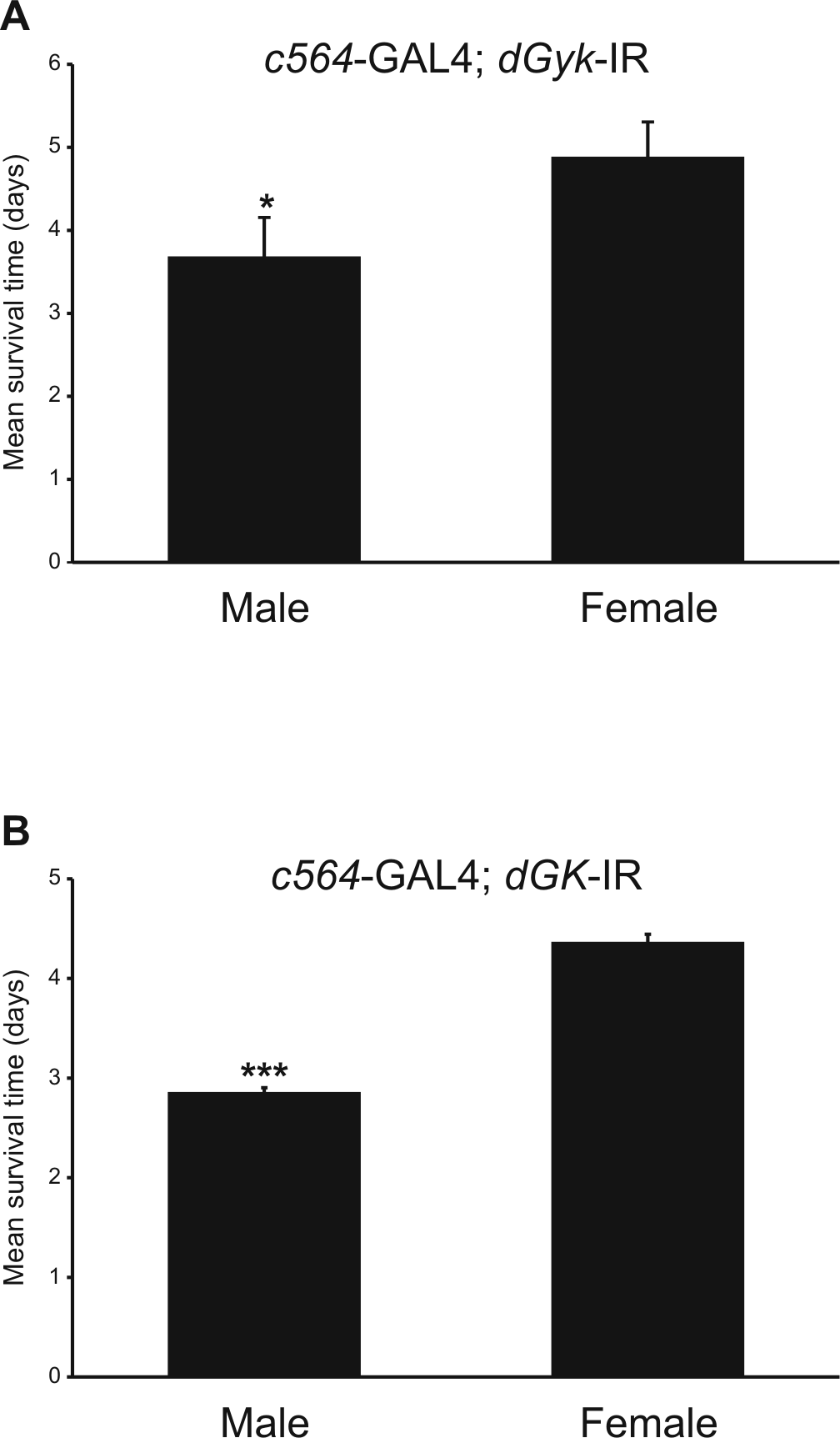

Supplement: Figure S5 — Glycerol hypersensitive sex differences. For RNAi knockdown flies, males were found to be more hypersensitive to glycerol than females. Glycerol hypersensitive survivorship assays were performed using single sex groups of flies. A) c564-GAL4; dGyk-IR adult flies on 1.5 M glycerol, 5% sucrose, 1.3% agarose. B) c564-GAL4; dGK-IR adult flies on 3 M glycerol, 5% sucrose, 1.3% agarose. Each assay used 8–10 day old flies, n>100. Survivorship curves were analyzed using a Log-rank test on the Kaplan and Meier data. * P<0.05, ***<0.001. (TIFF) [file pone.0031779.s005.tif]

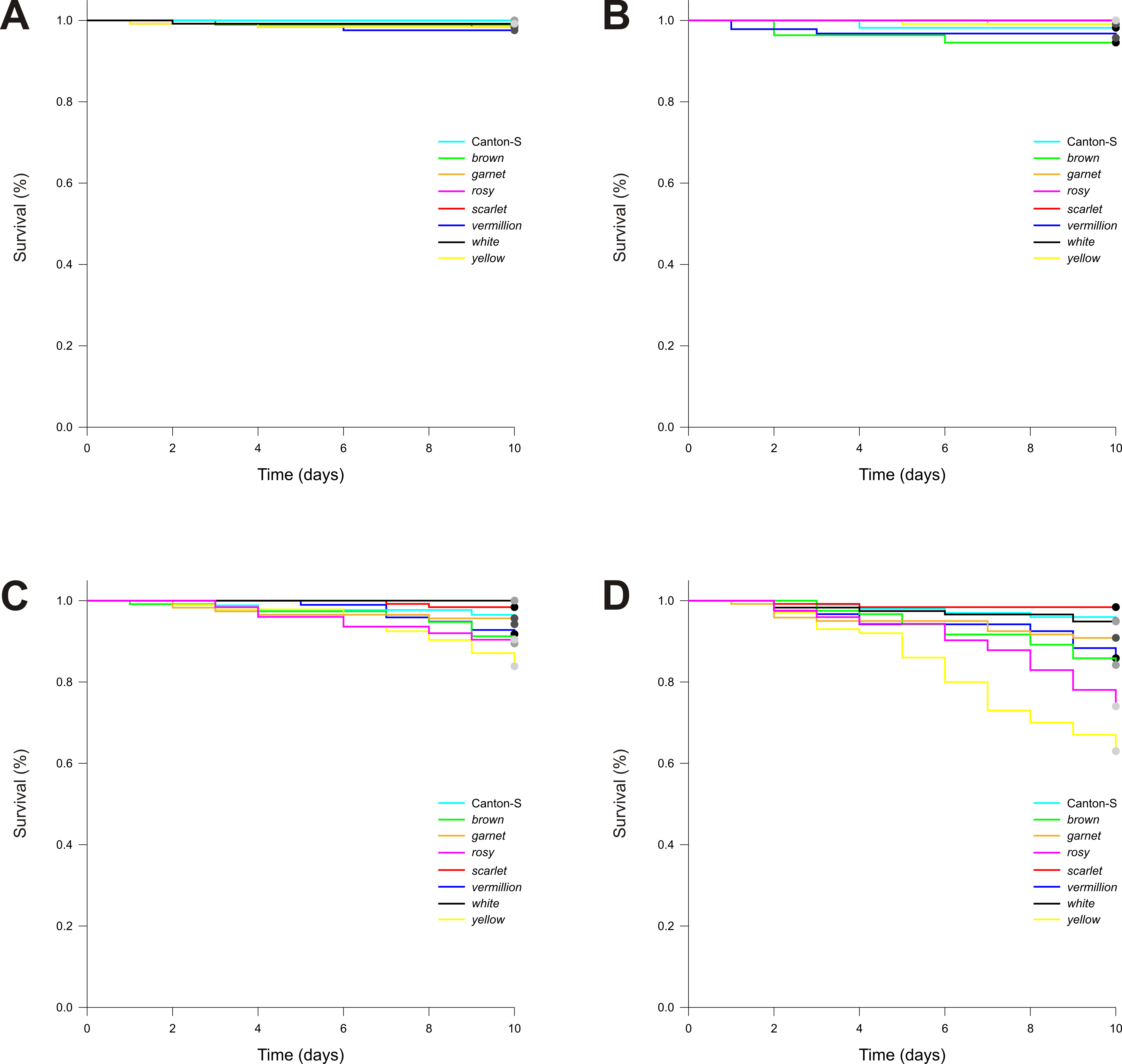

Supplement: Figure S6 — Control survivorship assays. Flies heterozygous for eye pigmentation null mutations in trans to A) c564-GAL4; dGyk-IR and B) c564-GAL4; dGK-IR are tolerant to a sucrose only food source over 10 days (5% sucrose, 1.3% agarose). C) Using a 2 M glycerol, 5% sucrose food source, heterozygous pigmentation null mutations in trans to the c564-GAL4 driver show some glycerol hypersensitivity after 10 days. D) Using a 3 M glycerol, 5% sucrose food source, heterozygous pigmentation null mutations in trans to the c564-GAL4 driver show increased glycerol hypersensitivity after 10 days compared to the 2 M glycerol 5% sucrose food source. In both C and D, control flies are more tolerant to glycerol than the c564-GAL4; dGyk-IR and c564-GAL4; dGK-IR knockdown flies (Figure 7). As a positive control, survivorship assays were performed using progeny from yellow flies, a mutant fly line previously shown to be desiccation sensitive. For each genotype, female flies (n>100) were aged 6–10 days on complete fly food before placing on the defined food source. (TIFF) [file pone.0031779.s006.tif]

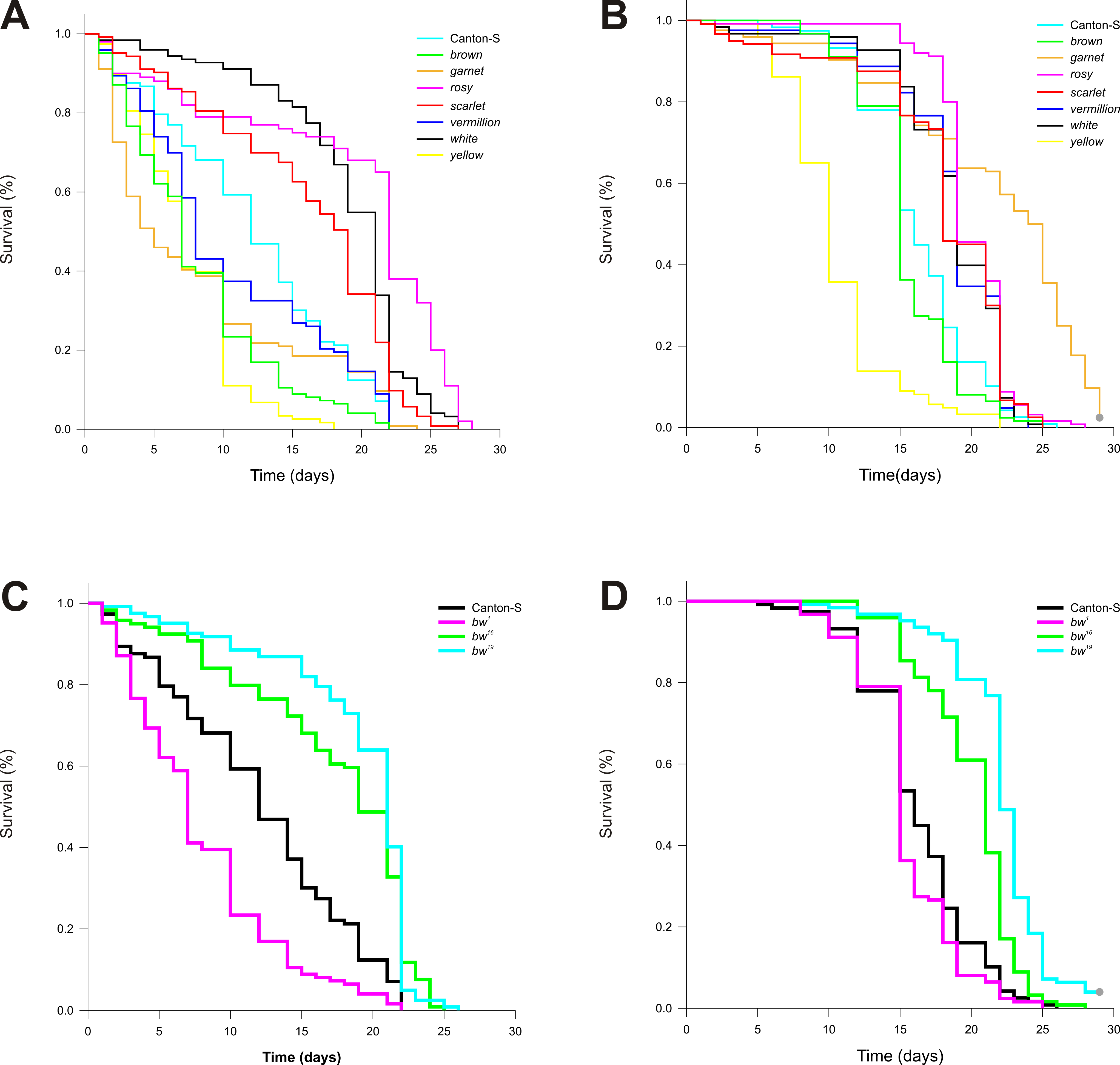

Supplement: Figure S7 — Survival analysis of pigmentation homozygous null mutant flies on defined food sources. A) 3 M glycerol, 5% sucrose food source, and B) 5% sucrose only food source. As a positive control, survivorship assays were performed using progeny from yellow flies, a mutant fly line previously shown to be desiccation sensitive. For each genotype, female flies (n>100) were aged 6–10 days on complete fly food before placing on the defined food source. Flies were counted every 24 hr until all were dead. (TIF) [file pone.0031779.s007.tif]
