# Supplementary material for: Glycerol Hypersensitivity in a Drosophila Model for Glycerol Kinase Deficiency Is Affected by Mutations in Eye Pigmentation Genes
Source: PLoS One. 2012 Mar 9;7(3):e31779. doi: 10.1371/journal.pone.0031779 (PMC3302884; doi:10.1371/journal.pone.0031779)
Supplement: Table S1 — Summary of RNAi, over-expression, and P element insertion fly lines. (DOC) [file pone.0031779.s008.doc]

| Fly line | Type | Construct | GAL4 driver | **GK activity** | **Glycerol hypersensitive?** | Comment |
| --- | --- | --- | --- | --- | --- | --- |
| *dGyk*-IR | RNAi | pUDs-GFP | *Tub*-GAL4 | reduced | Yes | - |
| *dGyk*-IRii | RNAi | pWIZ | *Tub*-GAL4 | reduced | Yes | target of RNAi overlaps to *dGyk*-IR |
| *dGK*-IR | RNAi | pUDs-GFP | *Tub*-GAL4 | reduced | Yes | - |
| *dGK*-IRii | RNAi | pWIZ | *Tub*-GAL4 | reduced | Yes | target of RNAi is non-overlapping to that of *dGK*-IR |
| *dGyk*-OE | over-expression | pEX-UAS | c564-GAL4 | not determined | No | - |
| *dGK*-OE | over-expression | pES-UAS | c564-GAL4 | not determined | No | embryonic lethal with *Tub*-GAL4 driver |
| *dGyk*00237 | P element insertion | piggyBac | NA | reduced | Yes | homozygous P element insertion in *dGyk* |

-

**Table S1.** Summary of RNAi, over-expression, and P element insertion fly lines.
