# Supplementary material for: Glycerol Hypersensitivity in a Drosophila Model for Glycerol Kinase Deficiency Is Affected by Mutations in Eye Pigmentation Genes
Source: PLoS One. 2012 Mar 9;7(3):e31779. doi: 10.1371/journal.pone.0031779 (PMC3302884; doi:10.1371/journal.pone.0031779)
Supplement: Table S2 — (DOC) [file pone.0031779.s009.doc]

| BDSC id. number | Day of 50% survival | | | | P lethal genotype |
| --- | --- | --- | --- | --- | --- |
|  | *dGyk*-RNAi screen | | *dGK*-RNAi screen | |  |
|  | male | female | male | female |  |
| 10183 | 4 | 3 | 4 | 6 | P{ry[+t7.2]=PZ}CrebA[03576] ry[506]/TM3, P{ry[+t7.2]=ftz/lacC}SC1, ry[RK] Sb[1] Ser[1] |
| 10184 | 2 | 4 | 3 | 6 | P{ry[+t7.2]=PZ}Abl[04674] ry[506]/TM3, ry[RK] Sb[1] Ser[1] |
| 10236 | 3 | 2 | 3 | 5 | P{ry[+t7.2]=PZ}ps[10615] ry[506]/TM3, ry[RK] Sb[1] Ser[1] |
| 10241 | 5 | 2 | 2 | 4 | P{ry[+t7.2]=PZ}l(3)rM060[rM060] ry[506]/TM3, ry[RK] Sb[1] Ser[1] |
| 10297 | 3 | 4 | 3 | 5 | ry[506] P{ry[+t7.2]=PZ}l(3)rN346[rN346]/TM3, ry[RK] Sb[1] Ser[1] |
| 10321 | 7 | 3 | 5 | 7 | ry[506] P{ry[+t7.2]=PZ}hh[rJ413] CG31457[rJ413]/TM3, ry[RK] Sb[1] Ser[1] |
| 10327 | 7 | 2 | 3 | 6 | ry[506] P{ry[+t7.2]=PZ}sec13[01031]/TM3, ry[RK] Sb[1] Ser[1] |
| 10343 | 2 | 4 | 5 | 5 | ry[506] P{ry[+t7.2]=PZ}Tl[rK344], l(3)rK344[rK344]/TM3, ry[RK] Sb[1] Ser[1] |
| 10344 | 5 | 2 | 4 | 5 | ry[506] P{ry[+t7.2]=PZ}sda[03884], l(3)03884[03884]/TM3, ry[RK] Sb[1] Ser[1] |
| 10345 | 6 | 4 | 3 | 5 | ry[506] P{ry[+t7.2]=PZ}btz[rL203]/TM3, ry[RK] Sb[1] Ser[1] |
| 10721 | 7 | 2 | 3 | 5 | P{ry[+t7.2]=PZ}CG31195[03806] CG17838[03806] ry[506]/TM3, ry[RK] Sb[1] Ser[1] |
| 11483 | 2 | 2 | 3 | 2 | ry[506] P{ry[+t7.2]=PZ}Fer2LCH[00035]/TM3, ry[RK] Sb[1] Ser[1] |
| 11486 | 3 | 2 | 2 | 4 | ry[506] P{ry[+t7.2]=PZ}osa[00090]/TM3, ry[RK] Sb[1] Ser[1] |
| 11487 | 2 | 3 | 2 | 5 | ry[506] P{ry[+t7.2]=PZ}Atg6[00096]/TM3, ry[RK] Sb[1] Ser[1] |
| 11490 | 2 | 3 | 3 | 5 | w[1118]; P{ry[+t7.2]=PZ}ppl[00217] AcCoAS[00217] ry[506]/TM3, Sb[1] Ser[1] |
| 11491 | 2 | 1 | 4 | 3 | P{ry[+t7.2]=PZ}Mo25[00274] ry[506]/TM3, ry[RK] Sb[1] Ser[1] |
| 11493 | 4 | 3 | 3 | 4 | ry[506] P{ry[+t7.2]=PZ}slmb[00295]/TM3, ry[RK] Sb[1] Ser[1] |
| 11494 | 2 | 3 | 3 | 3 | w[1118]; P{ry[+t7.2]=PZ}Atg1[00305] ry[506]/TM3, Sb[1] Ser[1] |
| 11497 | 3 | 4 | 3 | 2 | ry[506] P{ry[+t7.2]=PZ}Fer1HCH[00451]/TM3, ry[RK] Sb[1] Ser[1] |
| 11498 | 3 | 3 | 3 | 2 | P{ry[+t7.2]=PZ}l(3)00506[00506] ry[506]/TM3, ry[RK] Sb[1] Ser[1] |
| 11500 | 9 | 3 | 2 | 2 | P{ry[+t7.2]=PZ}l(3)00534[00534] ry[506]/TM3, ry[RK] Sb[1] Ser[1] |
| 11501 | 3 | 3 | 3 | 3 | P{ry[+t7.2]=PZ}stv[00543] ry[506]/TM3, ry[RK] Sb[1] Ser[1] |
| 11505 | 2 | 2 | 3 | 3 | ry[506] P{ry[+t7.2]=PZ}l(3)00643[00643]/TM6B, Tb[1] |
| 11509 | 6 | 3 | 2 | 2 | ry[506] P{ry[+t7.2]=PZ}l(3)00720[00720]/TM3, ry[RK] Sb[1] Ser[1] |
| 11510 | 4 | 2 | 2 | 2 | P{ry[+t7.2]=PZ}Aats-ile[00827] ry[506]/TM3, ry[RK] Sb[1] Ser[1] |
| 11511 | 3 | 3 | 3 | 4 | P{ry[+t7.2]=PZ}l(3)00835[00835] ry[506]/TM3, ry[RK] Sb[1] Ser[1] |
| 11513 | 8 | 7 | 3 | 2 | ry[506] P{ry[+t7.2]=PZ}tmod[00848]/TM3, ry[RK] Sb[1] Ser[1] |
| 11514 | 7 | 5 | 3 | 4 | P{ry[+t7.2]=PZ}l(3)00864[00864] ry[506]/TM3, ry[RK] Sb[1] Ser[1] |
| 11515 | 2 | 3 | 3 | 3 | P{ry[+t7.2]=PZ}osp[00865]; ry[506] P{PZ}zfh1[00865]/TM3, ry[RK] Sb[1] Ser[1] |
| 11518 | 3 | 2 | 3 | 4 | P{ry[+t7.2]=PZ}l(3)01029[01029] P{PZ}l(3)87Df[01029] ry[506]/TM3, ry[RK] Sb[1] Ser[1] |
| 11520 | 7 | 3 | 2 | 5 | P{ry[+t7.2]=PZ}Rm62[01086] ry[506]/TM3, ry[RK] Sb[1] Ser[1] |
| 11522 | 3 | 3 | 3 | 3 | ry[506] P{ry[+t7.2]=PZ}Hmgcr[01152]/TM3, ry[RK] Sb[1] Ser[1] |
| 11524 | 3 | 2 | 2 | 3 | ry[506] P{ry[+t7.2]=PZ}ssh[01207]/TM3, ry[RK] Sb[1] Ser[1] |
| 11527 | 6 | 2 | 3 | 3 | P{ry[+t7.2]=PZ}lab[01241] ry[506]/TM3, ry[RK] Sb[1] Ser[1] |
| 11529 | 2 | 2 | 3 | 3 | P{ry[+t7.2]=PZ}Snr1[01319] ry[506]/TM3, ry[RK] Sb[1] Ser[1] |
| 11531 | 3 | 3 | 3 | 4 | ry[506] P{ry[+t7.2]=PZ}cpo[01432]/TM3, ry[RK] Sb[1] Ser[1] |
| 11533 | 2 | 3 | * | * | ry[506] P{ry[+t7.2]=PZ}Atpalpha[01453a] P{PZ}stg[01453b]/TM3, ry[RK] Sb[1] Ser[1] |
| 11536 | 2 | 2 | 2 | 3 | P{ry[+t7.2]=PZ}Syx13[01470] ry[506]/TM3, ry[RK] Sb[1] Ser[1] |
| 11537 | 2 | 3 | 2 | 4 | P{ry[+t7.2]=PZ}RpLP0[01544] ry[506]/TM3, ry[RK] Sb[1] Ser[1] |
| 11538 | 1 | 3 | 3 | 2 | ry[506] P{ry[+t7.2]=PZ}srp[01549]/TM3, ry[RK] Sb[1] Ser[1] |
| 11539 | 3 | 3 | 2 | 4 | ry[506] P{ry[+t7.2]=PZ}blp[01618]/TM3, ry[RK] Sb[1] Ser[1] |
| 11540 | 9 | 8 | 3 | 4 | P{ry[+t7.2]=PZ}l(3)01629[01629] ry[506]/TM3, ry[RK] Sb[1] Ser[1] |
| 11541 | 1 | 3 | 4 | 2 | P{ry[+t7.2]=PZ}Bre1[01640] ry[506]/TM6B, ry[CB] Tb[+] |
| 11542 | 2 | 4 | 2 | 3 | tap[01658] P{ry[+t7.2]=PZ}blot[01658] ry[506]/TM3, ry[RK] Sb[1] Ser[1] |
| 11543 | 3 | 5 | 2 | 4 | P{ry[+t7.2]=PZ}polo[01673] ry[506]/TM3, ry[RK] Sb[1] Ser[1] |
| 11545 | 3 | 4 | 3 | 4 | P{ry[+t7.2]=PZ}01705a; ry[506] P{PZ}Doa[01705b]/TM3, ry[RK] Sb[1] Ser[1] |
| 11548 | 3 | 5 | 3 | 3 | P{ry[+t7.2]=PZ}simj[01814] ry[506]/TM3, ry[RK] Sb[1] Ser[1] |
| 11552 | 2 | 3 | 2 | 4 | ry[506] P{ry[+t7.2]=PZ}l(3)01969[01969]/TM3, ry[RK] Sb[1] Ser[1] |
| 11554 | 4 | 3 | 3 | 2 | P{ry[+t7.2]=PZ}l(3)02094[02094] ry[506]/TM3, ry[RK] Sb[1] Ser[1] |
| 11557 | 2 | 3 | 3 | 3 | w[1118]; ry[506] P{ry[+t7.2]=PZ}ppan[02231]/TM3, Sb[1] Ser[1] |
| 11558 | 4 | 3 | 2 | 5 | P{ry[+t7.2]=PZ}fry[02240] ry[506]/TM3, ry[RK] Sb[1] Ser[1] |
| 11560 | 2 | 4 | 4 | 6 | P{ry[+t7.2]=PZ}Xe7[02248] ry[506]/TM3, ry[RK] Sb[1] Ser[1] |
| 11561 | 4 | 2 | 2 | 5 | P{ry[+t7.2]=PZ}Hph[02255] ry[506]/TM3, ry[RK] Sb[1] Ser[1] |
| 11562 | 1 | 3 | 2 | 4 | P{ry[+t7.2]=PZ}Aly[02267] ry[506]/TM3, ry[RK] Sb[1] Ser[1] |
| 11563 | 2 | 3 | 2 | 5 | P{ry[+t7.2]=PZ}Baldspot[02281] ry[506]/TM3, ry[RK] Sb[1] Ser[1] |
| 11564 | 2 | 4 | 2 | 3 | ry[506] P{ry[+t7.2]=PZ}Tm1[02299]/TM3, ry[RK] Sb[1] Ser[1] |
| 11565 | 2 | 2 | 2 | 4 | P{ry[+t7.2]=PZ}l(3)02331[02331] ry[506]/TM3, ry[RK] Sb[1] Ser[1] |
| 11567 | 9 | 3 | 3 | 3 | ry[506] P{ry[+t7.2]=PZ}l(3)neo42[02404]/TM3, ry[RK] Sb[1] Ser[1] |
| 11568 | 2 | 2 | 2 | 3 | P{ry[+t7.2]=PZ}tws[02414] ry[506]/TM3, ry[RK] Sb[1] Ser[1] |
| 11569 | 3 | 2 | 2 | 5 | ry[506] P{ry[+t7.2]=PZ}l(3)02515[02515]/TM3, ry[RK] Sb[1] Ser[1] |
| 11570 | 2 | 2 | 2 | 4 | ry[506] P{ry[+t7.2]=PZ}l(3)02521[02521]/TM3, ry[RK] Sb[1] Ser[1] |
| 11571 | 2 | 2 | 3 | 3 | P{ry[+t7.2]=PZ}l(3)73Ah[02540] ry[506]/TM3, ry[RK] Sb[1] Ser[1] |
| 11572 | 3 | 6 | 3 | 5 | P{ry[+t7.2]=PZ}frc[02619] ry[506]/TM3, ry[RK] Sb[1] Ser[1] |
| 11574 | 2 | 2 | 3 | 5 | P{ry[+t7.2]=PZ}l(3)02640[02640] ry[506] |
| 11575 | 4 | 3 | 3 | 4 | ry[506] P{ry[+t7.2]=PZ}ttk[02667]/TM3, ry[RK] Sb[1] Ser[1] |
| 11577 | 4 | 4 | 3 | 5 | P{ry[+t7.2]=PZ}l(3)02732[02732] ry[506]/TM3, ry[RK] Sb[1] Ser[1] |
| 11584 | 2 | 2 | 3 | 5 | P{ry[+t7.2]=PZ}Hem[03335] ry[506]/TM3, ry[RK] Sb[1] Ser[1] |
| 11585 | 5 | 2 | 3 | 5 | P{ry[+t7.2]=PZ}gpp[03342] ry[506]/TM3, ry[RK] Sb[1] Ser[1] |
| 11586 | 4 | 2 | 3 | 5 | ry[506] P{ry[+t7.2]=PZ}l(3)03346[03346]/TM3, ry[RK] Sb[1] Ser[1] |
| 11589 | 1 | 1 | 3 | 5 | ry[506] P{ry[+t7.2]=PZ}heph[03429]/TM3, ry[RK] Sb[1] Ser[1] |
| 11590 | 3 | 2 | 3 | 4 | P{ry[+t7.2]=PZ}CtBP[03463] ry[506]/TM3, ry[RK] Sb[1] Ser[1] |
| 11595 | 3 | 3 | 3 | 5 | P{ry[+t7.2]=PZ}Aats-trp[03559] ry[506]/TM3, ry[RK] Sb[1] Ser[1] |
| 11597 | 1 | 2 | 2 | 4 | P{ry[+t7.2]=PZ}l(3)03644[03644] ry[506]/TM3, ry[RK] Sb[1] Ser[1] |
| 11602 | 10 | 2 | 3 | 4 | P{ry[+t7.2]=PZ}Tom34[03692] ry[506]/TM3, ry[RK] Sb[1] Ser[1] |
| 11603 | 4 | 9 | 3 | 5 | P{ry[+t7.2]=PZ}v(3)03699[03699] ry[506] |
| 11604 | 10 | 2 | 3 | 4 | ry[506] P{ry[+t7.2]=PZ}repo[03702]/TM3, ry[RK] Sb[1] Ser[1] |
| 11616 | 10 | 3 | 3 | 3 | P{ry[+t7.2]=PZ}CycA[03946] ry[506]/TM3, ry[RK] Sb[1] Ser[1] |
| 15451 | Not tested | Not tested | * | * | y[1] w[67c23]; P{w[+mC] y[+mDint2]=EPgy2}Atpalpha[EY05545]/TM3, Sb[1] Ser[1] |
