# Supplementary material for: Glycerol Hypersensitivity in a Drosophila Model for Glycerol Kinase Deficiency Is Affected by Mutations in Eye Pigmentation Genes
Source: PLoS One. 2012 Mar 9;7(3):e31779. doi: 10.1371/journal.pone.0031779 (PMC3302884; doi:10.1371/journal.pone.0031779)
Supplement: Methods S1 — Western Blotting. (DOCX) [file pone.0031779.s010.docx]

**Supplemental Methods**

**Western blotting**

Protein was extracted from groups of six 3^rd^ instar larvae in extraction buffer (1% KCl; 1 mM EDTA) and protease inhibitor (Roche). Quantification was performed using Bio-Rad protein quantification reagent and 10 ug run on an 8% acrylamide gel. After transfer onto invitrolon™ PVDF membrane (Invitrogen) the membrane was blocked in 10% non-fat milk in PBS plus 0.05% tween (Bio-Rad). The primary polyclonal antibody to GFP (Clontech; #632459) was hybridized overnight at 4°C (1:5000). The secondary HRP-conjugated goat anti-rabbit IgG antibody (Bio-rad; #170-6515) was incubated for 1 hr at room temperature (1:10,000). Signal detection was performed using the ECL plus Western blotting detection reagent kit (Amersham™; GE Healthcare) and exposure to X-ray film (Kodak biomax light film). Western blots were then re-probed using a rabbit beta-actin antibody (Cell Signaling Technology; #4967) overnight at 4°C (1:3000) followed by incubation with HRP-conjugated goat anti-rabbit IgG antibody (Bio-rad; #170-6515) for 1 hr at room temperature (1:10,000).
